# Dithieno[3,2‐*b*:2ʹ,3ʹ‐*d*]pyrrol‐Fused Asymmetrical Electron Acceptors: A Study into the Effects of Nitrogen‐Functionalization on Reducing Nonradiative Recombination Loss and Dipole Moment on Morphology

**DOI:** 10.1002/advs.201902657

**Published:** 2020-01-19

**Authors:** Wei Gao, Tao Liu, Rui Sun, Guangye Zhang, Yiqun Xiao, Ruijie Ma, Cheng Zhong, Xinhui Lu, Jie Min, He Yan, Chuluo Yang

**Affiliations:** ^1^ Shenzhen Key Laboratory of Polymer Science and Technology College of Materials Science and Engineering Shenzhen University Shenzhen 518060 P. R. China; ^2^ Department of Chemistry Hubei Key Lab on Organic and Polymeric Optoelectronic Materials Wuhan University Wuhan 430072 P. R. China; ^3^ Department of Chemistry and Hong Kong Branch of Chinese National Engineering Research Center for Tissue Restoration and Reconstruction Hong Kong University of Science and Technology Clear Water Bay Kowloon 999077 Hong Kong; ^4^ The Institute for Advanced Studies Wuhan University Wuhan 430072 P. R. China; ^5^ eFlexPV Limited (China) Shenzhen 518000 P. R. China; ^6^ Department of Physics Chinese University of Hong Kong New Territories 999077 Hong Kong

**Keywords:** asymmetrical, dipole moment, energy loss, nonfullerene acceptors, organic solar cells

## Abstract

Energy loss (*E*_loss_) consisting of radiative recombination loss (Δ*E*_1_ and Δ*E*_2_) and nonradiative recombination loss (Δ*E*_3_) is considered as an important factor for organic solar cells (OSCs). Herein, two *N*‐functionalized asymmetrical small molecule acceptors (SMAs), namely N7IT and N8IT are designed and synthesized, to explore the effect of N on reducing *E*_loss_ with sulfur (S) as a comparison. N7IT‐based OSCs achieve not only a higher PCE (13.8%), but also a much lower *E*_loss_ (0.57 eV) than those of the analogue (a‐IT)‐based OSCs (PCE of 11.5% and *E*_loss_ of 0.72 eV), which are mainly attributed to N7IT's significantly enhanced charge carrier density (promoting *J*_SC_) and largely suppressed nonradiative *E*_loss_ by over 0.1 eV (enhancing *V*_OC_). In comparison, N8IT, with an extended π‐conjugated length, shows relatively lower photovoltaic performance than N7IT (but higher than a‐IT) due to the less favorable morphology caused by the excessively large dipole moment of the asymmetrical molecule. Finally, this work sheds light on the structure–property relationship of the *N*‐functionalization, particularly on its effects on reducing the *E*_loss_, which could inspire the community to design and synthesize more *N*‐functionalized SMAs.

## Graphical abstract

By designing *N*‐functionalized asymmetrical acceptors N7IT and N8IT, the effects of nitrogen (N) atom on reducing nonradiative recombination loss (Δ*E*_3_) and dipole moment on morphology are revealed.

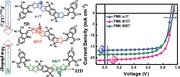

## 1 Introduction

Owing to their advantages in fabricating lightweight, flexible and semitransparent photovoltaic panels through cost‐effective printing methods, bulk‐heterojunction (BHJ) organic solar cells (OSCs) have shown great prospects for the smart‐city and indoor applications.^1^, ^2^, ^3^ The past two decades have witnessed the rapid increase of the power conversion efficiency (PCE) of OSCs with the main contribution from the innovation of photoactive materials,^4^, ^5^, ^6^, ^7^, ^8^, ^9^, ^10^ device optimization,^11^, ^12^ and interface engineering.^13^, ^14^ The breakthroughs in the material design and synthesis of small molecule acceptors (SMAs) has seen impressive progresses since 2015, opening a new landscape for OSCs.^15^ The state‐of‐the‐art SMAs are mainly based on an acceptor‐donor‐acceptor (A‐D‐A) structure, e.g., the fused‐ring electron acceptors (FREAs), with ITIC as the most representative example. ITIC‐like SMAs are ideal examples showing the combination of an electron‐rich fused‐ring core, electron‐deficient end groups, and outstretched side chains. Merits of such type of SMAs lie in the easily tunable chemical structures that allows judicious optimization of their optical properties,^16^, ^17^, ^18^ lowest unoccupied molecular orbital (LUMO)/highest occupied molecular orbital (HOMO) levels^19^, ^20^, ^21^ and crystallization/aggregation properties.^22^, ^23^, ^24^, ^25^, ^26^, ^27^, ^28^, ^29^ As a result, the recent polymer:SMA combination, i.e., PM6:Y6, shows over 16% PCEs in single‐junction devices, which greatly advances the process of OSCs.^30^, ^31^, ^32^

The enhancement of PCE is inseparable from the growing understanding of the structure–property relationship for the active layer materials. From a chemistry point of view, the structures of donor and acceptor materials can be simply considered as a combination of a carbon skeleton and a set of functional atoms that are connected by single, double or triple bonds. These functional elements including sulfur (S), oxygen (O), nitrogen (N), selenium (Se), silicon (Si), and halogens (F, Cl, and Br) play important roles in adjusting the photoelectric properties of materials and realizing energy conversion from sunlight to electricity.^33^, ^34^ For example, S‐containing heteroaromatics (thiophene and thieno[3,2‐*b*]thiophene) show small steric effects and strong quinone resonance structures, and could form intramolecular and intermolecular noncovalent interaction (S–S, S–O, and S–F) to stabilize molecular conformation and enhance π–π stacking.^34^ Highly electronegative halogen atoms are able to tune the absorption and energy levels, promote carrier mobility, and regulate the blend morphology of the active layer.^35^, ^36^, ^37^ Nitrogen atom, possessing a lone pair of electrons and three covalent bonds, exhibits a strong electron‐donating ability and provides an ideal site for sidechain engineering. Except acting as electron‐donating atom in carbazole and dithieno[3,2‐*b*:2ʹ,3ʹ‐*d*]pyrrol like groups, N as a functional element is more likely to be incorporated into electron‐withdrawing groups via forming imine, imide, amide, and cyano structures. Those N‐containing building blocks include benzothiadiazole (BT),^38^ benzotriazole (BTz),^39^ perylene diimide (PDI),^40^ naphthalene diimide (NDI),^41^ thiophene pyrrolidone (TPD),^42^ diketopyrrolopyrrole (DPP),^43^ isoindigo (IID),^44^ and 2‐(3‐oxo‐2,3‐dihydro‐1*H*‐inden‐1‐ylidene)malononitrile (INIC),^15^ which have been widely utilized to construct organic semiconductor materials. Polymer donors based on these *N*‐functionalized moieties achieve over 11% PCE when blending with fullerene acceptors.^5^ Recently, Tang's group reported dithieno[3,2‐*b*:2ʹ,3ʹ‐*d*]pyrrol (DTP) as a good donor block for constructing a novel *N*‐functionalized core, namely 5,5,12,12‐tetrakis(4‐hexylphenyl)indacenobis‐(dithieno[3,2‐*b*:2′,3′‐*d*]pyrrol) (INP).^45^ Thanks to the electron‐rich N atom, INP‐based SMAs (INPIC‐4F) showed a 1.39 eV optical bandgap (*E*_g_^opt^) and achieved a PCE over 13% along with a low energy loss (0.54 eV, calculated from *E*_loss_ = *E*_g_^opt^ − *eV*_OC_, where *V*_OC_ is open‐circuit voltage and *E*_g_^opt^ is obtained from the edge of film absorption). Subsequently, Zou's group designed and synthesized another *N*‐functionalized fused‐ring core with a strong electron‐deficient BT unit embraced in.^46^ The electron‐rich nature of the N atom in combination with the BT unit endows Y6 with not only a redshifted absorption (edge over 900 nm), but also deep HOMO/LUMO energy levels. In combination with PM6 (polymer donor), Y6‐based device delivered an impressive PCE of 15.7% accompanied with a 25.3 mA cm^−2^ short‐circuit current (*J*_SC_) and a 0.50 eV *E*_loss_. These results demonstrate the importance and potential of N for constructing high‐performance SMAs. Therefore, gaining deeper insights into the role of N atom is beneficial for designing future SMAs.

In this study, we designed and synthesized two novel *N*‐functionalized SMAs, namely N7IT and N8IT, through incorporating a DTP donating block. Our recent works have shown the benefits of the asymmetrical structures including: i) it allows different functional groups to be modified on both wings of the FREAs, which enables a finer adjustment of basic properties, and ii) it can promote the π–π stacking and LUMO energy level due to the favorable form of stacking induced by the dipole moment and diversified and stable molecular conformations.^22^, ^23^, ^47^, ^48^ Different from enhancing the electron‐donating ability via enlarging the π‐conjunction of the central core (usually resulting in a redshifted absorption and a downshifted LUMO level), the N atom provides the core with a richer p‐electron and pushes the LUMO level up compared to the S‐containing analog (a‐IT). As a result, N7IT and N8IT feature a 1.42 eV *E*_g_^opt^ and a high‐lying LUMO level (−3.90 eV). OSCs based on PM6:N7IT achieve a high PCE of 13.8% with a 0.932 V *V*_OC_, a 21.04 mA cm^−2^ *J*_SC_, and a 70.5% fill factor (FF), significantly higher than the PM6:N8IT‐based OSC, which is in turn higher than the PM6:a‐IT‐based OSC. We investigate the energy loss by employing the Fourier‐transform photocurrent spectroscopy external quantum efficiency (FTPS‐EQE) method, which shows that the PM6:N7IT‐based device has an *E*_loss_ of 0.57 eV, 0.14 eV lower than that of the PM6:a‐IT device. The difference is mainly attributed to the considerably suppressed nonradiative recombination loss (0.370 eV vs 0.266 eV), which is beneficial to enhance the *V*_OC_. Moreover, transient photovoltage (TPV) and charge extraction (CE) experiments demonstrate that the *N*‐functionalized N7IT‐based device has a higher charge carrier density than the a‐IT‐based devices, which is beneficial for *J*_SC_ enhancement. These results illuminate that the N atom can be powerful in reducing *E*_loss_ while achieving a high PCE, which should encourage further development of the *N*‐functionalized asymmetrical FREAs.

## 2 Results and Discussion

### 2.1 Synthesis and Characterization

**Scheme 1:**
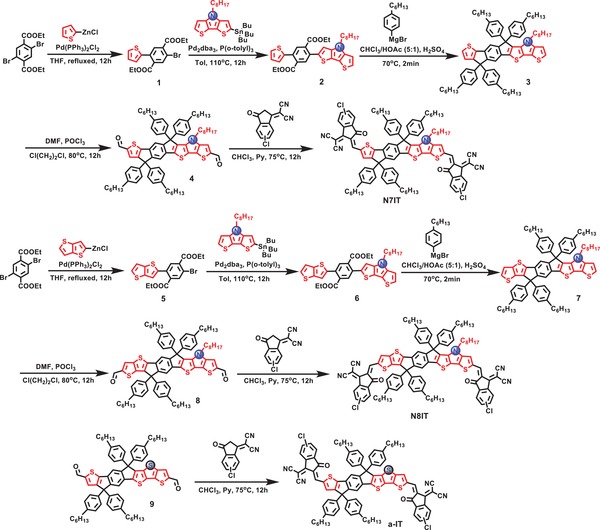
The synthetic routes and molecular structures of *N*‐containing N7IT and N8IT.

**Scheme** **1** displays the synthetic routes to our designed asymmetrical SMAs N7IT and N8IT. The key asymmetrical intermediate 2 (or 6) flanking a thiophene (or thieno[3,2‐*b*]thiophene) and DTP units in different wings were synthesized through a stepwise approach successively involving a Negishi and a Stille coupling reaction. Then, excessive (4‐hexylphenyl)magnesium bromide was used to accomplish the conversion of turning the ester groups of compound 2 (or 6) into hydroxyls which were subjected to an acid‐mediated intramolecular Friedel–Crafts cyclization reaction to produce compound 3 (or 7). It should be noted that the time of Friedel–Crafts reaction should be strictly controlled, otherwise the product cannot be obtained effectively. Subsequently, two formyl groups were successfully introduced into compound 3 (or 7), which was followed by an end‐capping step with 2‐(5/6‐chloro‐3‐oxo‐2,3‐dihydro‐1*H*‐inden‐1‐ylidene)malononitrile (IC‐Cl) to receive the target SMAs N7IT (or N8IT). Meanwhile, an asymmetrical control molecule a‐IT was also synthesized to investigate the function of the N atom. The synthetic details and characterization data including ^1^H/^13^C NMR and MS are presented in the Supporting Information. Three SMAs all exhibit good solubility in commonly used solvents, which meets the essential requirements for the spin‐coating process.

### 2.2 Absorption and Energy Levels

**Figure 1:**
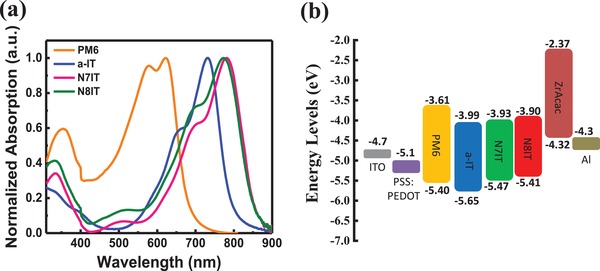
a) Normalized UV–vis absorption spectra of a‐IT, N7IT, and N8IT in thin films. b) Energy levels of materials involved in OSCs device.

The UV–vis absorption spectra of a‐IT, N7IT, and N8IT in dilute solution, thin films, and blend films (with PM6) were measured and shown in **Figure** **1**a and Figure S1 (Supporting Information), respectively, with the characteristic data listed in **Table** **1**. In solution, a‐IT, N7IT, and N8IT exhibit maximum absorption peaks of 691, 736, and 740 nm along with molar extinction coefficient being 1.40 × 10^5^, 2.53 × 10^5^, and 3.11 × 10^5^ m ^−1^ cm^−1^, respectively, indicating N atom can significantly enhance the absorption in both range and intensity. When coming to film state, a large redshift can be observed for all three SMAs attributed to strong intermolecular aggregation. The *E*_g_^opt^s calculated form absorption edges were found to be 1.54, 1.42, and 1.42 eV for a‐IT, N7IT, and N8IT neat films, respectively. Cyclic voltammetry (CV) measurements of PM6 and three SMAs were carried out, and their CV curves were plotted in Figure S2 (Supporting Information). According to the oxidation/reduction potentials, the LUMO/HOMO energy levels of PM6, a‐IT, N7IT, and N8IT were estimated to be −3.61/−5.40, −3.99/−5.65, −3.93/−5.47, and −3.90/−5.41 eV, respectively. Changing S atom into N atom obviously promotes the HOMO energy levels of N7IT and N8IT, and meanwhile slightly promote the LUMO energy levels, which largely lower the bandgap of SMAs. It is known that the driving force for exciton dissociation is determined by the energy offset (Δ*E*_offset_) between HOMO (or LUMO) energy levels of donor and acceptor. As the Δ*E*_offset_s of LUMO energy levels is enough for three blend systems, the small Δ*E*_offset_s of HOMO energy levels (0.07 eV for PM6:N7IT and 0.01 eV for PM6:N8IT) will have effects on effective exciton dissociation (to achieve large *J*_sc_). However, PM6 is still a suitable donor to match with N7IT and N8IT (for high *V*_oc_ and good morphology) because nearly zero Δ*E*_offset_ can also offer enough driving force for efficient exciton dissociation in nonfullerene acceptors‐based OSCs according to recent research.^49^ These positive effects of N atom on SMAs can be anticipated to realize a high *V*_OC_ and a large *J*_SC_ in one cell.

**Table 1:** Basic properties of three SMAs

Acceptor	λ_max_ ^a)^ [nm]	λ_onset_ ^a)^ [nm]	ε_max_ ^b)^ [m ^−1^ cm^−1^]	λ_max_ ^b)^ [nm]	λ_onset_ ^b)^ [nm]	*E*_g_^opt^ ^c)^ [eV]	LUMO^d)^ [eV]	HOMO^d)^ [eV]	LUMO^e)^ [eV]	HOMO^e)^ [eV]
a‐IT	691	746	1.40 × 10^5^	733	804	1.54	−3.99	−5.65	−3.63	−5.99
N7IT	736	792	2.53 × 10^5^	781	873	1.42	−3.93	−5.47	−3.55	−5.83
N8IT	740	798	3.11 × 10^5^	773	871	1.42	−3.90	−5.41	−3.54	−5.77

^a)^ In chloroform solution

^b)^ In a neat film

^c)^ Calculated from the empirical formula: *E*_g_^opt^ = 1240/λ_onset_

^d)^ CV method by measuring film in acetonitrile

^e)^ Obtained from DFT calculations.

### 2.3 DFT Calculations

To illuminate the effects of N as a functional element in SMAs, the density functional theory (DFT) calculation was performed where the long alkyl sidechains are simplified into methyl groups (Figure S3, Supporting Information). A small dihedral angle between donor core and end groups is revealed in a‐IT, N7IT and N8IT, and the near distance makes it a possibility that S atom from thiophene end in donating core can form noncovalent interactions with O atom from IC‐Cl. The intramolecular S–O conformational lock offers three SMAs a planar structure to promote π–π stacking in the solid state. The LUMO/HOMO energy levels of a‐IT, N7IT, and N8IT (Table 1) are calculated to be −3.63/−5.99, −3.55/−5.83, and −3.54/−5.77 eV, respectively, and the calculated absorption peaks are 623, 640, and 653 nm, respectively, which are consistent with the trend of measured results. Extending π‐conjugation length by incorporating electron‐donating block into fused‐ring core can increase the degree of delocalization of p‐electrons, which enhances the intramolecular charge transfer (ICT) state of SMAs and thus achieves redshifted absorption range. However, the LUMO level will decrease due to the easier charge transfer from core to end group. Replacing S atom with N atom does not increase the π‐conjugation length, but the lone pair electrons and stronger electron‐donating ability of N atom can not only promote ICT state but also increase the electron density of SMAs, which affords double effects of widening absorption and pushing HOMO/LUMO up. Moreover, the enhanced charge density will increase the excitation probability of ground state electrons and improve the ability to capture more solar photons, which results in higher molar extinction coefficients of *N*‐containing SMAs. These positive influences of N atom on SMAs are beneficial to simultaneously achieve enhanced *V*_OC_ and *J*_SC_.

### 2.4 Photovoltaic Performance

**Figure 2:**
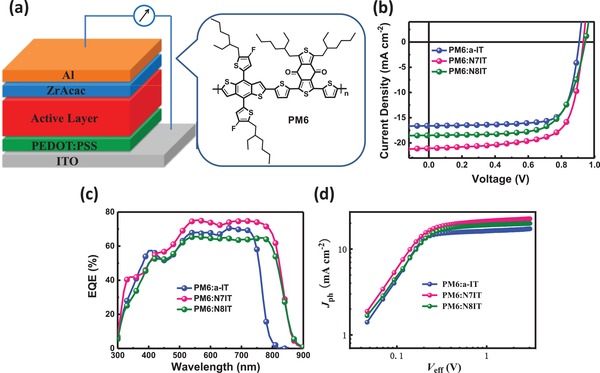
a) The structures of device and polymer donor PM6. b) The *J*–*V* curves of the optimal device. c) Corresponding EQE spectra. d) *J*_ph_ depends on *V*_eff_.

To investigate the potential of *N*‐functionalized SMAs involving in OSCs, we fabricated a series of conventional devices whose structure is shown as indium tin oxide (ITO)/poly(3,4‐ethylenedioxythiophene):poly(styrenesulfonate)(PEDOT:PSS)/PM6:acceptor (a‐IT, N7IT, or N8IT) /zirconium acetylacetonate (ZrAcac)/Al (**Figure** **2**a), where ITO and Al work as the anode and cathode, respectively, PEDOT:PSS and ZrAcac act as the hole transport layer (HTL) and cathode interfacial layer (CIL), respectively. Considering suitable energy levels and complementary absorption range, PM6 was selected as an ideal donor polymer to blend with three SMAs. Based on our previous device manufacturing experience, chloroform was utilized as the processing solvent which can well dissolve two component of donor and acceptor for fully mixing. The total concentration for blended solution is set as 16 mg mL^−1^ with an optimal D:A weight ratio of 1:1. In addition, 0.25% 1,8‐diiodooctane (DIO) during solution preparation and annealing at 100 °C after film formation were necessarily needed to realize optimal devices. For detailed fabrication methods and processes refer to the Supporting Information. The characteristic current density–voltage (*J*–*V*) curves of best‐performing OSCs and the corresponding external quantum efficiency (EQE) spectra are shown in Figure 2b,c, respectively, and the key photovoltaic parameters are summarized in **Table** **2**. As shown, OSCs based on PM6:N7IT achieved a *V*_OC_ of 0.932 V, a *J*_SC_ of 21.04 mA cm^−2^, and a FF of 70.5%, yielding a PCE as high as 13.82% (highest PCE for asymmetrical SMAs‐based OSCs), while PM6:N8IT‐based OSCs delivered an obviously lower PCE of 11.92% multiplying from a *V*_OC_ of 0.942 V, a *J*_SC_ of 18.53 mA cm^−2^, and a FF of 68.2%, suggesting N7IT is superior to N8IT mainly due to the morphology difference caused by dipole moment which will be discussed in detail blow. It is worth mentioning that N8IT owing a longer π‐conjugation than N7IT but observes a higher *V*_OC_ in devices, well confirming our previous researches on asymmetrical SMAs.^47^, ^48^ The photovoltaic performance of OSCs based on PM6:a‐IT were also investigated to make a comparison with PM6:N7IT‐based OSCs. Replacing S with N atom results in a dramatical enhancement of PCE from 11.46% to 13.82% accompanying with *V*_OC_ increasing from 0.907 to 0.932 V and *J*_SC_ increasing from 16.60 to 21.04 mA cm^−2^. Such results benefit from the unique merits of N in broadening absorption and elevating LUMO energy level, illuminating *N*‐containing SMAs is able to reduce *E*_loss_ of OSCs compared to their S‐containing analogue. In addition, device repeatability was confirmed by calculating the average values and mean square errors of PCEs from 20 devices, which are 11.13% ± 0.19%, 13.44% ± 0.26%, and 11.55% ± 0.28% for PM6:a‐IT‐, PM6:N7IT‐, and PM6:N8IT‐based OSCs, respectively. OSCs based on N7IT show an obviously broader photon response extending to 900 nm and a stronger intensity within a range from 400 to 800 nm compared with a‐IT‐based OSCs, which takes the responsibility for great promotion of *J*_SC_. It is observed that N8IT‐based OSCs have the same response range with N7IT‐based OSCs but a low value in the whole EQE spectra, attributed to unbalanced charge transport and unfavorable morphology (discussed below). The EQE‐integrated *J*_SC_s of OSCs based on a‐IT, N7IT, and N8IT are 16.06, 20.46, and 18.11 mA cm^−2^, respectively, which are consistent with the measured *J*_SC_s within an error of ≈3%.

**Table 2:** Photovoltaic parameters of studied OSCs

Active layer^a)^	*V*_oc_ ^b)^ [V]	*J*_sc_ ^c)^ [mA cm^−2^]	FF [%]	PCE^b)^ [%]
PM6:a‐IT	0.907(0.901 ± 0.004)	16.60(16.06)	76.2	11.46 (11.13 ± 0.19)
PM6:N7IT	0.932(0.925 ± 0.005)	21.04(20.46)	70.5	13.82 (13.44 ± 0.26)
PM6:N8IT	0.943(0.936 ± 0.007)	18.53(18.11)	68.2	11.92 (11.55 ± 0.28)

^a)^ Effective area of the device is 5.9 mm^2^

^b)^ Inside the brackets are the mean and mean square error of 20 devices

^c)^ Inside the brackets is the EQE‐integrated *J*_SC_.

### 2.5 Charge Carrier Density and Recombination

**Figure 3:**
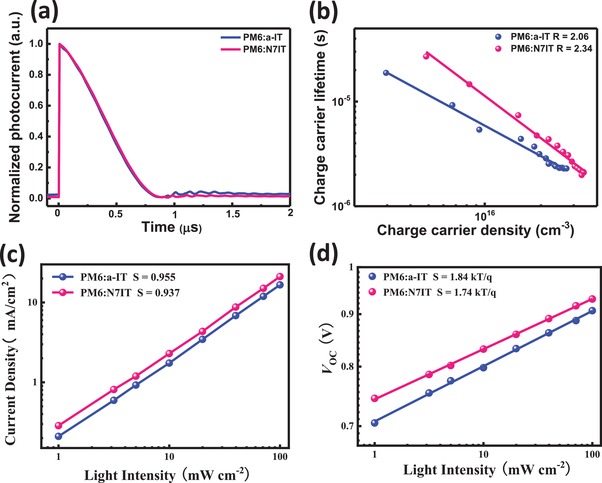
a) TPC measurements of PM6:a‐IT‐ and PM6:N7IT‐based optimal OSCs. b) Charge carrier lifetime as a function of charge carrier density, the solid lines represent linear fits of the data. c) *J*_sc_ dependence on light intensity. d) *V*_oc_ dependence on light intensity.

To explore the significantly higher *J*_SC_ of N7IT‐based devices, the charge extraction process in optimal OSCs based on a‐IT and N7IT was investigated by conducting transient photocurrent (TPC) measurements. As revealed in **Figure** **3**a, both devices exhibit nearly the same and short charge extraction time, suggesting efficient charge extraction from the BHJ layer. So, we further utilized the transient photovoltage and charge extraction techniques to detect the charge carrier density (*n*), and the charge carrier lifetimes measured by TPV (calculated from Figure S4, Supporting Information) as a function of charge carrier density obtained from CE (calculated from Figure S5, Supporting Information) is plotted in Figure 3b. It is found that OSCs with N7IT as acceptor own larger charge carrier density than that of a‐IT‐based OSCs at different charge carrier lifetimes, suggesting N atom can significantly promote the charge carrier density within devices. Under the same extraction conditions, the higher the charge carrier density, the more electrons or holes are obtained by the electrode at the same time, thus N7IT‐based OSCs obtained a higher *J*_SC_. However, higher charge carrier density means more serious recombination. To understand the degree of recombination in two devices, we determined the nongeminate recombination order *R* (*R* = λ + 1), which can be calculated via the equation τ = τ_0_(*n*_0_/*n*)*^λ^*, where τ_0_ and *n*_0_ are constants and λ is the so‐called recombination exponent. As calculated, *R* is 2.06 for a‐IT‐based devices and 2.34 for N7IT‐based devices.

In order to further understand the charge recombination mechanism in PM6:a‐IT and PM6:N7IT‐based OSCs, *J*_sc_ and *V*_oc_ under different light intensity (*P*) were measured. And the *J*_sc_ or *V*_oc_ dependence on light intensity curves are shown in Figure 3c,d, respectively. Generally, the slope of *V*_oc_ versus *P* in natural logarithm (*V*_oc_ – ln(*P*)) should be equal to *kT*/*q* if bimolecular recombination is the unique loss mechanism. When Shockley–Read–Hall (SRH) recombination, a trap‐assisted recombination created by interfacial defects and/or impurities in materials, was involved, the *V*_oc_ will exhibit stronger dependence on light intensity with a larger slope than *kT*/*q* (smaller than 2 *kT*/*q*).^50^ The *V*_oc_ dependence on *P* shows slopes (*S*) of 1.84 *kT*/*q* for PM6:a‐IT‐based device and 1.74 *kT*/*q* for PM6:N7IT‐based device, indicating the monomolecular (geminate) and bimolecular recombination coexist in two OSCs, and the monomolecular recombination mechanism was dominated. In addition, the slope (*S*) for bimolecular recombination is 0.955 and 0.937 for PM6:a‐IT‐ and PM6:N7IT‐based OSCs, respectively. Though N7IT‐based OSCs exhibits stronger recombination than that in a‐IT‐based OSCs, the difference of recombination order is not very big, which will not have a big impact on the *J*_SC_ boost.

### 2.6 Energy Loss

**Figure 4:**
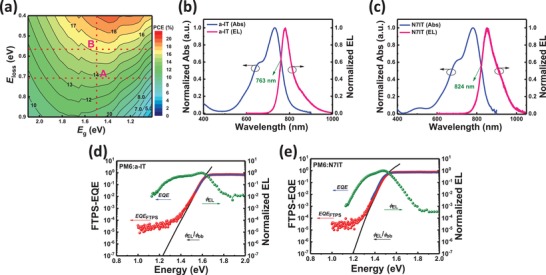
a) Contour map of PCE versus *E*_g_ and *E*_loss_. Normalized UV–vis absorption and EL spectra for b) a‐IT and c) N7IT neat films. Measured EQE (blue line), FTPS‐EQE (red line), EL (green line), and external quantum efficiency (black lines) of OSCs based on d) PM6:a‐IT and e) PM6:N7IT. The external quantum efficiency is determined by EL and the blackbody emission (ϕbb).

As revealed by the Shockley–Queisser (SQ) model that the PCE limit of OSCs is mainly restricted by the *E*_g_s of the involved photoactive materials and the *E*_loss_ of the cell, and **Figure** **4**a draws a contour map of PCE versus *E*_g_ and *E*_loss_. From this, we can find that it is impossible to break the PCE limit through only adjusting *E*_g_ when *E*_loss_ of a system is given. Though reducing *E*_loss_ is the most effective way to realize PCE breakthroughs (from point A to B, Figure 4a), the large *E*_loss_ of an OSC originated from unique physical properties of organic semiconductor remains a critical issue. In general, the *E*_loss_ during exciton separation and charge recombination processes consists of three sources specifying as the following equation^54^, ^55^

(1) $$E_{\text{loss}}=\left(E_{\text{gap}}-qV_{\text{oc}}^{\text{SQ}}\right)+\left(q\Delta V_{\text{oc}}^{\text{rad}}\right)+\left(q\Delta V_{\text{oc}}^{\text{nonrad}}\right)=\Delta E_{1}+\Delta E_{2}+\Delta E_{3}$$

where *E*_gap_ is the lower bandgap between a donor and an acceptor; *q* is the elementary charge; *V*_oc_^SQ^ is the maximum voltage by the Shockley–Queisser limit, where the EQE is assumed to be step‐wise; Δ*V*_oc_^rad^ is the voltage loss of radiative recombination from the absorption below the bandgap; Δ*V*_oc_^nonrad^ is the voltage loss of nonradiative recombination. ∆*E*_1_ is due to the radiative recombination originating from the absorption above the bandgap, which is an unavoidable *E*_loss_ in any types of solar cells typically within 0.25–0.30 eV. ∆*E*_2_ is due to the additional radiative recombination loss from the absorption below the bandgap (the absorption from charge transfer (CT) states due to the existence of driving energy contributed greatly to ∆*E*_2_). With the emergence of FREAs, the ∆*E*_2_ is nearly negligible because the *E*_offset_ (HOMO energy levels difference between donor and acceptor materials) of less than 0.1 eV can also offer enough driving force for efficient exciton dissociation. Leaved ∆*E*_3_, i.e., nonradiative recombination loss, as a large component (0.26–0.48 eV in SMAs‐based OSCs) of *E*_loss_, is critically important for further enhancing the *V*_OC_ and thus PCE of OSCs. Therefore, reducing *E*_loss_ is to suppress nonradiative *E*_loss_ (∆*E*_3_) through reasonable molecular design.

Electroluminescence (EL) measurements of a‐IT (Figure 4b) and N7IT (Figure 4c) neat films were carried out to find out the intersection with their absorption spectra, which are 763 nm for a‐IT and 824 nm for N7IT with *E*_gap_ of 1.625 and 1.505 eV, respectively. *E*_loss_ is defined as the difference between *E*_gap_ and *qV*_OC_ and calculated to be 0.718 and 0.573 eV for a‐IT‐ and N7IT‐based OSCs. A more than 20% *E*_loss_ drop via substituting a S atom in a‐IT with N atom reveals a magical power of N element in reducing *E*_loss_. To acquire more accurate *E*_loss_ values, the absorption and EL spectra of blend films were also measured (Figure S6 and Table S1, Supporting Information). The results showed the same trend of *E*_loss_ reduction (by 0.136 eV) with those measured in neat films by changing S atom in a‐IT with N atom. And the *E*_loss_ of PM6:N8IT‐based OSCs was found to be 0.588 eV which is slightly lower than that of PM6:N7IT‐based OSCs (0.595 eV). The Further investigations of which part of the *E*_loss_ is inhibited were done by performing Fourier‐transform photocurrent spectroscopy external quantum efficiency and EL spectra measurements (Figure 4d,e) with the extracted parameters summarized in **Table** **3**. $V_{\text{oc}}^{\text{SQ}}$ in Equation (1) is determined by *E*_CT_/*q* where *E*_CT_ represents the energy of CT state. *E*_CT_ can be estimated by fitting the lower energy part of EQE spectra based on the Marcus theory (Equation (2))^56^, ^57^

(2) $${\text{EQE}}_{\text{PV}}\left(E\right)=\frac{f}{E\sqrt{4\pi \lambda kT}}\text{exp}\left(\frac{-{\left(E_{\text{CT}}+\lambda -E\right)}^{2}}{4\lambda kT}\right)$$

where *k* is the Boltzmann constant, *T* is absolute temperature, λ is the reorganization energy, and *f* is the oscillator absorption strength, proportional to the donor/acceptor interface area. $V_{\text{oc}}^{\text{SQ}}\text{s}$ of a‐IT‐ and N7IT‐based OSCs were calculated to be 1.351 and 1.238 V corresponding to ∆*E*_1_ of 0.274 and 0.267 eV, respectively. ∆*E*_2_ reduced from ∆*E*_CT_ (an energy loss during charge generation) are 0.074 eV for a‐IT system and 0.040 eV for N7IT system. ∆*E*_3_ can be obtained by subtracting $V_{\text{oc}}^{\text{rad}}$ from the measured *V*_OC_, found to be 0.370 and 0.266 eV for a‐IT‐ and N7IT‐based devices, respectively. These results indicate that N atom has a significant effect in reducing *E*_loss_ of FREAs‐based OSCs by simultaneously suppressing ∆*E*_1,_ ∆*E*_2_ and ∆*E*_3_, especially ∆*E*_3_.

**Table 3:** Summary of parameters extracted from FTPS‐EQE and EL measurements

Active layer	*E*_gap_ [eV]	*V*_oc_ [V]	*E*_loss_ [eV]	$V_{\text{oc}}^{\text{SQ}}$ [V]	$\Delta E_{1}=E_{\text{gap}}-qV_{\text{oc}}^{\text{rad}}$ [eV]	$V_{\text{oc}}^{\text{rad}}$ [V]	$\Delta E_{2}=q\Delta V_{\text{oc}}^{\text{rad}}$ [eV]	$\Delta E_{3}=q\Delta V_{\text{oc}}^{\text{nonrad}}$ [eV]
PM6:a‐IT	1.625	0.907	0.718	1.351	0.274	1.277	0.074	0.370
PM6:N7IT	1.505	0.932	0.573	1.238	0.267	1.198	0.040	0.266
PM6:ITC‐2Cl^a)^	1.58	0.91	0.67	1.42	0.16	1.233	0.187	0.323
PM6:ITCPTC^a)^	1.65	0.95	0.70	1.54	0.11	1.309	0.231	0.359
PM6:IT‐4F^a)^	1.60	0.87	0.73	1.38	0.22	1.236	0.144	0.366
PM6:IT‐4Cl^a)^	1.56	0.80	0.76	1.28	0.28	1.207	0.073	0.470
PCE10:FOIC^b)^	1.38	0.741	0.64	1.11	0.27	1.069	0.041	0.329
PBDB‐T:Y1^c)^	1.44	0.87	0.57	1.12	0.27	1.12	0.05	0.25
PBDB‐T:Y2^c)^	1.40	0.82	0.57	1.09	0.27	1.09	0.04	0.26

^a)^ In the ref. 51

^b)^ In the ref. 52

^c)^ In the ref. 53.

**Figure 5:**
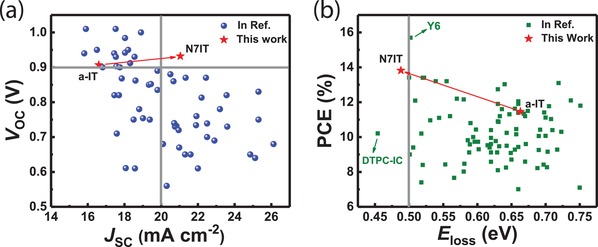
a) Scatter plot of *V*_OC_ versus *J*_SC_. b) Scatter plot of PCE versus *E*_loss_.

To further clarify the potential of N7IT, we compared N7IT with other reported high‐performance acceptors such as ITC‐2Cl, IT‐4F, FOIC and Y series in *E*_loss_, ∆*E*_1_, ∆*E*_2_ and ∆*E*_3_ measured by FTPS‐EQE and EL (Table 3, available data are rare due to the limitations of FTPS‐EQE test). It can be found that N7IT‐based devices yielded a very low *E*_loss_ (equaling to Y series‐based OSCs) among these systems with ∆*E*_2_ and ∆*E*_3_ significantly lower, suggesting *N*‐containing asymmetrical N7IT is a potential SMAs. Adding Cl atom into the end group of ITCPTC to form ITC‐2Cl lows the *E*_loss_ of cell by 0.07 eV and meanwhile suppress ∆*E*_3_ by 0.036 eV,^49^ whose effects are not more obvious than the effect that substituting S atom with N atom of donor core (from a‐IT to N7IT exhibits reduction of 0.145 eV in *E*_loss_ and 0.104 eV in ∆*E*_3_). Sun and co‐workers recently reported a ternary doping strategy to suppress ∆*E*_3_ of PCE10:FOIC system by 0.058 eV, which provides a good approach to reduce ∆*E*_3_.^49^ In contrast, the N atom has better effects in reducing ∆*E*_3_. To make a close comparison, we draw the scatter plot of *V*_OC_ versus *J*_SC_ based on reported results (Table S2, Supporting Information). As shown in **Figure** **5**a, it is not difficult to find that when an OSC reaches an over 0.9 V *V*_OC_, it is difficult to achieve a *J*_SC_ of more than 20 mA cm^−2^ or vice versa. However, PM6:N7IT‐based OSCs achieves such a breakthrough and stands in the area most difficult to reach. The scatter plot of PCE versus *E*_loss_ is also depicted, where *E*_loss_ is defined as the difference between *E*_g_^opt^ and *qV*_OC_. The data reveals that *E*_loss_ below 0.5 eV is very difficult to be achieved in single‐junction OSCs even for Y6 system. Our N7IT‐based devices realized a 0.488 eV *E*_loss_, ranking second in the existing known data. The above results all indicate that N7IT can achieve low *E*_loss_ among SMAs.

### 2.7 Exciton Dissociation and Carrier Mobility

Since the role of the N atom in reducing *E*_loss_ has been clarified, we now turn to figure out the reasons why N7IT performs better than N8IT. Two important processes of exciton dissociation and charge extraction were studied by plotting photocurrent (*J*_ph_) against effective applied voltage (*V*_eff_) (Figure 2d). As revealed, the *J*_ph_ can quickly reach saturation (*J*_sat_) at a low reversal voltage (within 0.5 V), indicating an efficient conversion process form photon to an electron in two SMAs‐based OSCs. The *J*_sat_ of N7IT‐ and N8IT‐based OSCs were found to be 22.52 and 19.90 mA cm^−2^, respectively. Maximum exciton generation rate (*G*_max_) related to *J*_sat_/*qL*, where *q* is the elementary charge and *L* is the thickness of the active layer (110 nm), were calculated to be 1.28 × 10^28^ m^−3^ s^−1^ for N7IT‐based OSCs and 1.13 × 10^28^ m^−3^ s^−1^ for N8IT‐based OSCs, indicating N7IT has a faster exciton generation rate than that of N8IT. The efficiencies of exciton dissociation (*P*_diss_) and charge collection (*P*_coll_), evaluated by the specific values of *J*_ph_/*J*_sat_ under short circuit and maximum power output condition, respectively, are 93.4/80.4% for N7IT devices and 93.1/74.8% for N8IT devices, respectively (Table S3, Supporting Information). It is not difficult to find that the *P*_coll_ of N8IT‐based OSCs is significantly lower than that of N7IT‐based OSCs, which takes major responsibility for the lower *J*_SC_ and FF in N8IT‐based OSCs. Moreover, the *P*_diss_ of PM6:a‐IT‐based OSCs is 96.5%, which is slightly higher than that of PM6:N7IT‐based OSCs, indicating that replacing S atom in a‐IT with N atom will slightly lower the *P*_diss_ of OSCs.

To further clarify the low *J*_SC_ and FF of N8IT‐based OSCs, the charge transport behaviors of N7IT and N8IT pure and blend films were surveyed by fabricating the electron‐only (ITO/ZnO/active layer/ZrAcac/Al) and hole‐only (ITO/MoO*_x_*/active layer/MoO*_x_*/Al) diodes. By fitting the data using the space charge limited current (SCLC) model (Figure S7, Supporting Information), we found the electron mobilities of N7IT and N8IT films are 7.21 × 10^−4^ and 5.92 × 10^−4^ cm^2^ V^−1^ s^−1^, respectively. It should be noted that the electron mobility of N7IT is significantly higher than that of a‐IT which was measured to be 4.32 × 10^−4^ cm^2^ V^−1^ s^−1^, indicating replacing S atom with N atom can enhance the electron mobility of SMAs. When donor was incorporated into, the hole/electron (*μ*_h_/*μ*_e_) mobilities become 7.01 × 10^−4^/4.31 × 10^−4^ cm^2^ V^−1^ s^−1^ for PM6:N7IT (*μ*_h_/*μ*_e_ = 1.63), and 6.92 × 10^−4^ /3.79 × 10^−4^ cm^2^ V^−1^ s^−1^ for PM6:N8IT (*μ*_h_/*μ*_e_ = 1.83). Compared to PM6:N7IT, PM6:N8IT achieves low and unbalanced charge transports, which is not beneficial to higher *J*_SC_ and FF.

### 2.8 Morphology

**Figure 6:**
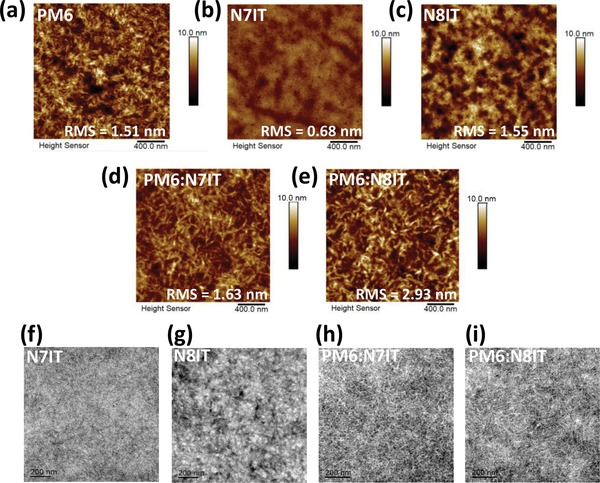
The AFM height sensor images for: a) a PM6 neat film; b) a N7IT neat film; c) a N8IT neat film; d) a PM6:N7IT blend film and e) a PM6:N8IT blend film. The TME images for: f) a N7IT neat film; g) a N8IT neat film; h) a PM6:N7IT blend film; i) a PM6:N8IT blend film.

Optimal conformation of N7IT predicted by DFT simulation is that two end groups are evenly distributed on both sides of the long axis of the molecule with intramolecular S–O interaction, while the optimal conformation for N8IT is changed with two end groups distributed on the same side of the long axis of the molecule (Figure S3, Supporting Information). Different conformation combined with the strong electron‐withdrawing ability of N atom endows N8IT a large inherent dipole moment of up to 12 Debye (5 Debye for N7IT) with its direction located in and parallel to the molecular plane. That means polar N8IT owns a pair of strong positive and negative charge centers within the molecule, which benefits to strengthen the intermolecular packing through Coulomb interactions. From atomic force microscope (AFM) measurements (**Figure** **6** and Figure S8: Supporting Information), it can be seen that the root‐mean‐square (RMS) roughness of N8IT film (1.55 nm) equals to that of PM6 film (1.51 nm) and is two times higher than that of N7IT film. In addition, the transmission electron microscope (TEM) measurements (Figure 6f,g) revealed that N8IT neat film shows obviously larger phase scale than that of N7IT neat film. Those results well indicate that N8IT possesses significantly stronger crystallization performance than N7IT. When adding two SMAs into PM6, the RMS roughness change for PM6:N7IT can be ignored and meanwhile a great interpenetrating nanofiber microstructure is developed, which can be clearly observed from TEM image (Figure 6h) and suggests a good mutual compatibility between PM6 and N7IT. However, a big RMS roughness change can be observed for PM6:N8IT and the nanofiber of PM6 is destroyed to form a lumpy morphology, which may be caused by a faster crystallization rate and stronger crystallization tendency of N8IT induced by its overlarge dipole moment. We guested the mechanism is N8IT phase pushing PM6 out to form large donor/acceptor phase during the crystallization process of film formation. The comparison between TEM images (Figure 6h,i) suggests a poor micromorphology of PM6:N8IT with larger and uneven phase separation relative to that of PM6:N7IT. Moreover, the rough surface of PM6:N8IT is not conducive to the contact between the active layer and electrodes and thus lowers the *P*_coll_ of PM6:N8IT‐based OSCs, resulting in a low *J*_SC_ and FF.

**Figure 7:**
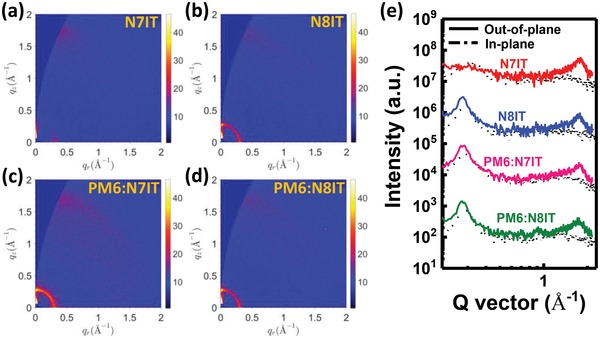
2D‐GIWAXS patterns: a) a N7IT neat film; b) a N8IT neat film; c) a PM6:N7IT blend film; d) a PM6:N8IT blend film, and e) corresponding cut‐line profiles.

N8IT has an extended π‐conjugated length and a significantly bigger dipole moment than those of N7IT, reasonably, N8IT should exhibit a redshifted absorption peak and enhanced electron mobility than those of N7IT in film, however, we got opposite results. We suspected the packing pattern of N8IT has altered. To figure out the reasons, the grazing‐incidence wide‐angle X‐ray scattering (GIWAXS) measurements were performed. The 2D‐GIWAXS patterns and corresponding cut‐line profiles of two SMAs ‐based neat and blend films are shown in **Figure** **7** with calculated parameters listed in Table S4 (Supporting Information) (out‐of‐plane, OOP) and Table S5 (Supporting Information) (in‐plane, IP). N7IT displays a well‐defined face‐on orientation with a strong (010) diffraction peak located at 1.75 Å^−1^ (*d*‐spacing: 3.58 Å), while N8IT reveals a coexisted face‐on and edge‐on orientations with both strong (010) and (100) diffraction peaks in OOP direction. It should be noted that the coherence length (CL) of (100) lamellar packing of N8IT in OOP direction (151 Å) is significantly larger than that in in‐plane (IP) direction (93 Å), indicating N8IT film has a greater trend to take an edge‐on orientation. Moreover, the (010) π–π stacking of N8IT in IP direction is negligible and its CL (18 Å) in OOP direction is smaller than that of N7IT (21 Å) in the same direction, suggesting a weaker π–π stacking and accounting for the lower electron mobility of N8IT film. In general, the larger the size of a molecule, the less likely it is to take an orientation perpendicular to the substrate (edge‐on) because it will take more energy to make it stand up. Our previous study in asymmetrical SMAs proved that such kind of molecule (with a dipole moment smaller than 7 Debye) is more likely to take a face‐on orientation. Thus, we deduced such a change may be caused by the overlarge dipole moment of N8IT, which may form some amorphous complexes relying on strong Coulomb forces. Since PM6 take a mixed orientation of face‐on and edge‐on in neat film (Figure S9, Supporting Information), these PM6:N7IT and PM6:N8IT blend film also show the same mixed orientations. But, the negative effect of overlarge dipole moment was also brought into PM6:N8IT blend film. The larger CL of (100) lamellar packing (142 Å) and smaller CL of (010) π–π stacking (11.7 Å) of PM6:N8IT in OOP direction than those of PM6:N7IT (125/16.4 Å) in OOP direction, indicating a stronger trend of taking an edge‐on orientation and a weaker π–π stacking in PM6:N8IT, which is consistent with the lower electron/charge mobilities of PM6:N8IT blend film and thus lower *J*_SC_ and FF.

## 3 Conclusion

In summary, we designed and synthesized two novel asymmetrical SMAs, namely N7IT and N8IT, by incorporating a DTP unit into the central core. Compared to the analog molecule (a‐IT), the *N*‐functionalized N7IT shows an over 50 nm red‐shifted absorption and a simultaneously elevated LUMO level. These features provide the N7IT‐based devices with higher *V*_OC_, *J*_SC_ and PCE (13.8%) than the a‐IT‐based OSCs. Furthermore, our characterizations show that substituting the S atom of dithieno[3,2‐*b*:2′,3′‐*d*]thiophene in a‐IT with the N atom not only enhances the charge carrier density, but also reduces the *E*_loss_ of the resultant OSCs via suppressing the nonradiative *E*_loss_ by over 0.1 eV. In contrast, the electron‐rich N combined with specific molecular conformation makes N8IT to show an excessively large dipole moment (up to 12 Debye,) which causes excessive molecular crystallization and forces the SMAs to adopt an orientation perpendicular to the substrate in the film‐state. The less favorable morphology leads to relatively reduced efficiency for N8IT‐based control devices. Overall, the results allow us to deeply understand the roles of N atom in reducing *E*_loss_ and regulating the morphology, which will guide future design of efficient SMAs.

## Conflict of Interest

The authors declare no conflict of interest.

## Supporting information

Supporting Information
