# Supplementary material for: Dithieno[3,2‐b:2ʹ,3ʹ‐d]pyrrol‐Fused Asymmetrical Electron Acceptors: A Study into the Effects of Nitrogen‐Functionalization on Reducing Nonradiative Recombination Loss and Dipole Moment on Morphology
Source: Adv Sci (Weinh). 2020 Jan 19;7(5):1902657. doi: 10.1002/advs.201902657 (PMC7055560; doi:10.1002/advs.201902657)
Supplement: Supplementary file 1 — Supporting Information [file ADVS-7-1902657-s001.pdf]

Copyright WILEY-VCH Verlag GmbH & Co. KGaA, 69469 Weinheim, Germany, 2020.

ADVANCED SCIENCE
Open Access

Supporting Information

for *Adv. Sci.,* DOI: 10.1002/advs.201902657

Dithieno[3,2-*b*:2',3'-*d*]pyrrol-Fused Asymmetrical Electron Acceptors: A Study into the Effects of Nitrogen-Functionalization on Reducing Nonradiative Recombination Loss and Dipole Moment on Morphology

Wei Gao, Tao Liu, Rui Sun, Guangye Zhang, Yiqun Xiao, Ruijie Ma, Cheng Zhong, Xinhui Lu,* Jie Min,* He Yan,* and Chuluo Yang**

Copyright WILEY-VCH Verlag GmbH & Co. KGaA, 69469 Weinheim, Germany, 2019.

Supporting Information

Dithieno[3,2-*b*:2′,3′-*d*]pyrrol-Fused Asymmetrical Electron Acceptors: A Study into the Effects of Nitrogen-Functionalization on Reducing Nonradiative Recombination Loss and Dipole Moment on Morphology

Wei Gao, Tao Liu, Rui Sun, Guangye Zhang, Yiqun Xiao, Ruijie Ma, Cheng Zhong, Xinhui Lu,* Jie Min,* He Yan*, and Chuluo Yang**

Dr. W. Gao and Prof. C. Yang
Shenzhen Key Laboratory of Polymer Science and Technology, College of Materials Science and Engineering, Shenzhen University, Shenzhen 518060, China
E-mail: clyang@whu.edu.cn
Dr. W. Gao, Dr. C. Zhong and Prof. C. Yang
Department of Chemistry, Hubei Key Lab on Organic and Polymeric Optoelectronic Materials, Wuhan University, Wuhan, 430072, People's Republic of China
Dr. T. Liu, R. Ma and Prof. H. Yan
Department of Chemistry and Hong Kong Branch of Chinese National Engineering Research Center for Tissue Restoration & Reconstruction, Hong Kong University of Science and Technology, Clear Water Bay, Kowloon, Hong Kong
E-mail: liutaozhx@ust.hk, hyan@ust.hk
R. Sun and Prof. J. Min
The Institute for Advanced Studies, Wuhan University, Wuhan 430072, China
E-mail: min.jie@whu.edu.cn
Y. Xiao and Prof. X. Lu
Department of Physics, Chinese University of Hong Kong, New Territories, Hong Kong
E-mail: xhlu@phy.cuhk.edu.hk
Dr. G. Zhang
eFlexPV Limited (China), Shenzhen 518060, China.

Synthesis

All solvents and reagents were used as received from commercial sources and used without further purification except for toluene and tetrahydrofuran (THF) which were dried by potassium sodium alloy under refluxing condition. Compounds diethyl 2,5-dibromoterephthalate, 1-bromo-4-hexylbenzene and polymer donor PM6 were purchased from commercial source. Compound **1**, Compound **5**, Compound **9**, 4-octyl-2-(tributylstannyl)-4*H*-dithieno[3,2-*b*:2',3'-*d*]pyrrole and IC-Cl were synthesized according to the reported methods.

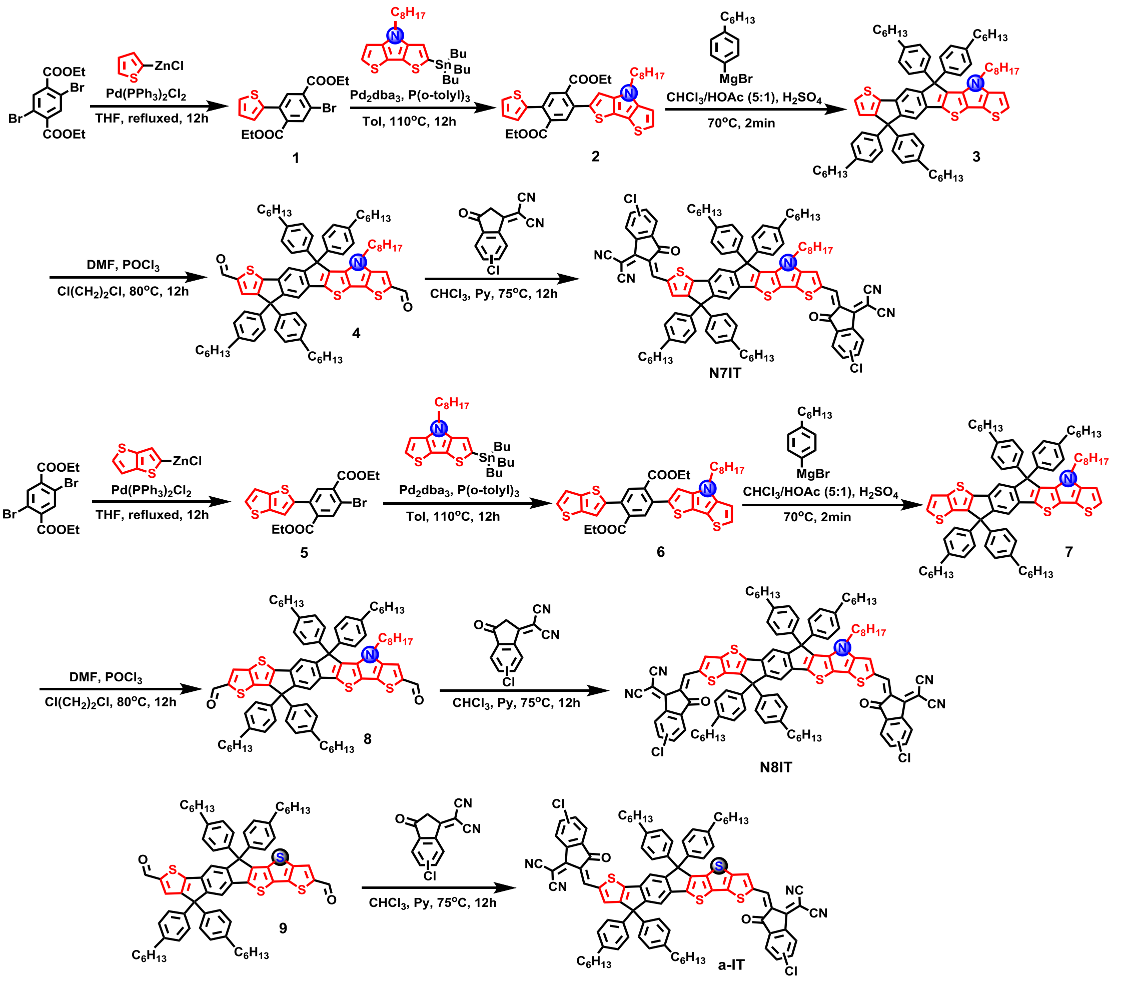

Scheme S1. The synthetic routes for N7IT, N8IT and a-IT.

Synthesis of ***compound 2***: To a dry 100 ml round-bottom flask, compound **1** (1.0 g, 2.61 mmol), 4-octyl-2-(tributylstannyl)-4*H*-dithieno[3,2-*b*:2',3'-*d*]pyrrole (1.59 g, 2.74 mmol), Pa_2dba_3 (36 mg, 0.04 mmol), P(*o*-tolyl)$_3$ (48 mg, 0.16 mmol) and anhydrous toluene (40 ml) was added under argon protection. Then, the mixture was refluxed at 110°C for 24h. After cooling to room temperature, the solvent was evaporated and the residue was purified by silicon chromatography using petroleum ether/dichloromethane (2:1, v/v) as eluent to get the product as a yellow oil (1.4 g, 90%). ^{1}H NMR (400 MHz, $CDCl_3$), δ (ppm): 7.90 (s, 1H), 7.78 (s, 1H), 7.39 (dd, J_1 = 1.2 Hz, J_2 = 4.8 Hz, 1H), 7.17 (d, J = 5.2 Hz, 1H), 7.07-7.11 (m, 3H), 7.01 (d, J = 5.6 Hz, 1H), 4.17-4.27 (m, 6H), 1.85-1.88 (m, 2H), 1.24-1.32 (m, 10H), 1.13-1.17 (m, 6H), 0.84-0.88 (m, 3H). ^{13}C NMR (100 MHz, CDCl3), δ (ppm): 168.29, 167.85, 144.82, 144.67, 140.61, 137.20, 134.16, 134.00, 133.97, 132.95, 131.70, 131.67, 127.39, 126.95, 126.47, 123.69, 114.74, 110.97, 110.94, 61.79, 61.70, 47.51, 31.82, 29.29, 29.18, 27.05, 22.64, 14.13, 13.98, 13.85. HRMS (ESI) m/z calcd for $C_{32}H_{36}NO_4S_3^+$ (M+H)$^+$ 594.18010, found 594.18030.

*Synthesis of **compound 3***: To a stirring solution of compound **2** (1.3 g, 2.19 mmol) in dry THF (50 ml) was added dropwise a solution of (4-hexylphenyl)magnesiumbromide which was prepared from 1-bromo-4-hexylbenzene (3.75 g, 15.5 mmol) and magnesium (410 mg, 17 mmol) at room temperature under argon atmosphere. After finishing adding, the solution was heated to reflux for 16 hours. After cooling to room temperature, the mixture was poured into water (150 ml) and extracted with ethyl acetate (3 × 100 ml), then washed with saturated salt water three times and dried over anhydrous Na_2SO_4. After removal of the solvent under reduced pressure, the brown crude product was obtained and then used in the next step without further purification. The crude product was dissolved in chloroform (100 ml) and glacial acetic acid (10 ml), and 1 ml concentrated sulfuric acid in 5 ml glacial acetic acid was dropwise into the solution, then the mixture was stirred at 70°C for 2 min. After cooling to room temperature, the mixture was extracted with dichloromethane and washed with water. The collected organic layer was dried over anhydrous Na_2SO_4 and concentrated. The residue was purified by column chromatography on silica gel using a mixture solvent as eluent (petroleum ether/dichloromethane, v/v = 20/1) to give a yellow solid (500 g, 20%). ^{1}H NMR (400 MHz, $CDCl_3$), δ (ppm): 7.41 (s, 1H), 7.35 (s, 1H), 7.33 (d, *J* = 8.4 Hz, 4H), 7.20 (d, *J* = 4.8 Hz, 1H), 7.16 (d, *J* = 8.4 Hz, 4H), 7.04-7.08 (m, 9H), 6.93 (d, *J* = 4.8 Hz, 1H), 6.89 (d, *J* = 5.2 Hz, 1H), 3.74 (t, *J* = 8.0 Hz, 2H), 2.56 (t, J = 7.6 Hz, 8H), 1.53-1.59 (m, 10H), 0.97-1.34 (m, 34H), 0.85-0.89 (m, 15H). ^{13}C NMR (100 MHz, $CDCl_3$), δ (ppm): 156.12, 155.52, 153.26, 144.27, 142.09, 141.65, 141.30, 139.60, 138.68, 135.78, 134.90, 128.74, 128.23, 127.90, 126.97, 123.09, 122.57, 117.51, 116.07, 116.01, 115.95, 111.48, 62.56, 62.36, 48.23, 35.56, 35.53, 31.89, 31.72, 31.71, 31.34, 31.30, 30.35, 29.31, 29.28, 29.16, 26.83, 22.66, 22.60, 14.11. HRMS (ESI) m/z calcd for $C_{76}H_{91}NS_3^+$ $(M)^+$ 1113.63082, found 1113.63000.

*Synthesis of **compound 4***: To a dry 100 mL two-necked round bottom flask, 10 ml anhydrous *N*, *N*-dimethylformamide (DMF) was added, and the solution was cooled to 0°C and stirred when 2 ml phosphorous oxychloride ($POCl_3$) was added by syringe under argon protection. The mixture was stirred at 0°C for 1 hour, and then compound **3** (490 mg, 0.44 mmol) in dry 1, 2-dichloroethane (20 ml) was added. Then, the mixture solution was allowed to reflux overnight. After cooling to room temperature, 100 ml water was added and the mixture was extracted with dichloromethane (DCM), and the organic layer was collected, washed with water and dried with anhydrous Na_2SO_4. After removal of the solvent under reduced pressure, the residue was purified by column chromatography on silica gel using a

mixture solvent as eluent (petroleum ether/dichloromethane, v/v = 1/1) to give an orange solid (422 mg, 82%). ^{1}H NMR (400 MHz, $CDCl_3$), δ (ppm): 9.84 (s, 1H), 9.79 (s, 1H), 7.62 (s, 1H), 7.55 (s, 1H), 7.52 (s, 1H), 7,44 (s, 1H), 7.31 (d, *J* = 8.0 Hz, 4H), 7.07-7.15 (m, 12H), 3.78 (t, *J* = 8.0 Hz, 2H), 2.58 (t, *J* = 7.6 Hz, 8H), 1.54-1.60 (m, 10H), 0.99-1.29 (m, 34H), 0.86-0.90 (m, 15H). ^{13}C NMR (100 MHz, $CDCl_3$), δ (ppm): 182.88, 182.80, 157.22, 156.21, 154.91, 151.17, 145.47, 145.23, 144.06, 143.64, 142.34, 142.07, 140.87, 140.28, 140.06, 137.62, 137.57, 134.39, 132.24, 128.59, 127.74, 124.49, 119.78, 118.60, 117.81, 116.78, 62.85, 62.56, 48.38, 35.57, 35.54, 31.90, 31.73, 32.72, 31.37, 31.30, 30.47, 29.33, 29.29, 29.19, 29.15, 26.85, 22.68, 22.62, 14.14, 14.13. HRMS (ESI) m/z calcd for $C_{78}H_{92}NO_2S_3^+$ $(M+H)^+$ 1170.62847, found 1170.62891.

*Synthesis of **N7IT***: To a 100 ml round bottom flask, compound **4** (150 mg, 0.128 mmol) and IC-Cl (117 mg, 0.51 mmol) were added under argon protection. Then, deoxidized chloroform (40 ml) was added and stirred for a while when pyridine (1 ml) was added. The mixture was kept stirring at 75°C for 12 h. After removal of chloroform of reaction mixture under reduced pressure, 100 ml methanol was added and the precipitate was collected by filtration. The residue was purified by column chromatography on silica gel using a mixture solvent as eluent (petroleum ether/dichloromethane, v/v = 1/1) to give a dark solid (175 mg, 86%). ^{1}H NMR (400 MHz, $CDCl_3$), δ (ppm): 8.86 (s, 2H), 8.53-8.63 (m, 2H), 7.76-7.86 (m, 2H), 7.50-7.69 (m, 6H), 7.32 (d, *J* = 8.0 Hz, 4H), 7.10-7.15 (m, 12H), 3.77 (s, 2H), 2.59 (t, *J* = 7.6 Hz, 8H), 1.60 (br, 10H), 1.01-1.30 (m, 34H), 0.87-0.91 (m, 15H). ^{13}C NMR (100 MHz, $CDCl_3$), δ (ppm): 187.22, 187.09, 159.33, 158.39, 157.66, 156.41, 150.41, 147.19, 142.72, 142.36, 141.19, 140.83, 140.34, 139.96, 138.66, 138.36, 136.97, 136.59, 135.38, 135.04, 128.76, 128.47, 127.74, 126.11, 123.78, 120.20, 118.76, 117.50, 62.91, 62.57, 48.55, 35.56, 31.90, 31.73, 31.71, 31.36, 31.30, 30.48, 29.38, 29.27, 29.18, 29.13, 26.81, 22.69, 22.61, 14.14. MALDI-TOF-MS m/z: [M] calcd. for $C_{102}H_{97}Cl_2N_5O_2S_3$, 1589.62, found 1589.11.

*Synthesis of **compound 6***: To a dry 100 ml round-bottom flask, compound **5** (1.0 g, 2.28 mmol), 4-octyl-2-(tributylstannyl)-4*H*-dithieno[3,2-*b*:2',3'-*d*]pyrrole (1.40 g, 2.39 mmol), Pa_2dba_3 (36 mg, 0.04 mmol), P(*o*-tolyl)$_3$ (48 mg, 0.16 mmol) and anhydrous toluene (40 ml) was added under argon protection. Then, the mixture was refluxed at 110°C for 24h. After cooling to room temperature, the solvent was evaporated and the residue was purified by silicon chromatography using petroleum ether/dichloromethane (2:1, v/v) as eluent to get the product as a yellow oil (1.30 g, 88%). ^{1}H NMR (400 MHz, $CDCl_3$), δ (ppm): 7.92 (s, 1H),

7.82 (s, 1H), 7.34 (d, *J* = 5.2 Hz, 1H), 7.28 (s, 1H), 7.27 (d, *J* = 5.2 Hz, 1H), 7.17 (d, *J* = 5.2 Hz, 1H), 7.08 (s, 1H), 7.01 (d, *J* = 5.6 Hz, 1H), 4.22-4.28 (m, 4H), 4.18 (t, *J* = 6.8 Hz, 2H), 1.85-1.88 (m, 2H), 1.24-1.31 (m, 10H), 1.15 (t, *J* = 7.2 Hz, 3H), 1.12 (t, *J* = 7.2 Hz, 3H), 0.86 (t, *J* = 6.4 Hz, 3H). ^{13}C NMR (100 MHz, CDCl3), δ (ppm): 168.21, 167.69, 144.90, 144.70, 142.28, 139.85, 139.39, 137.08, 134.46, 134.04, 133.94, 132.98, 131.78, 127.32, 123.79, 119.46, 119.19, 115.98, 114.77, 111.08, 110.94, 61.83, 61.80, 47.51, 31.82, 31.63, 30.44, 29.29, 29.18, 27.06, 22.70, 22.65, 14.19, 14.13, 13.99, 13.83. HRMS (ESI) m/z calcd for $C_{34}H_{36}NO_4S_4^+$ $(M+H)^+$ 650.15217, found 650.15271.

Synthesis of ***compound 7***: To a stirring solution of compound **6** (1.2 g, 1.85 mmol) in dry THF (50 ml) was added dropwise a solution of (4-hexylphenyl)magnesiumbromide which was prepared from 1-bromo-4-hexylbenzene (3.75 g, 15.5 mmol) and magnesium (410 mg, 17 mmol) at room temperature under argon atmosphere. After finishing adding, the solution was heated to reflux for 16 hours. After cooling to room temperature, the mixture was poured into water (150 ml) and extracted with ethyl acetate (3 × 100 ml), then washed with saturated salt water three times and dried over anhydrous Na_2SO_4. After removal of the solvent under reduced pressure, the brown crude product was obtained and then used in the next step without further purification. The crude product was dissolved in chloroform (100 ml) and glacial acetic acid (10 ml), and 1 ml concentrated sulfuric acid in 5 ml glacial acetic acid was dropwise into the solution, then the mixture was stirred at 70°C for 2 min. After cooling to room temperature, the mixture was extracted with dichloromethane and washed with water. The collected organic layer was dried over anhydrous Na_2SO_4 and concentrated. The residue was purified by column chromatography on silica gel using a mixture solvent as eluent (petroleum ether/dichloromethane, v/v = 20/1) to give a yellow solid (433 g, 20%). ^{1}H NMR (400 MHz, $CDCl_3$), δ (ppm): 7.42 (s, 1H), 7.41 (s, 1H), 7.35 (d, *J* = 8.4 Hz, 4H), 7.24 (d, *J* = 5.2 Hz, 1H), 7.22 (d, *J* = 5.2 Hz, 1H), 7.17 (d, *J* = 8.4 Hz, 4H), 7.05-7.09 (m, 9H), 6.89 (d, *J* = 5.2 Hz, 1H), 3.72 (t, *J* = 8.0 Hz, 2H), 2.52-2.57 (m, 10H), 1.53-1.61 (m, 8H), 1.27-1.36 (m, 34H), 0.82-0.90 (m, 15H). ^{13}C NMR (100 MHz, $CDCl_3$), δ (ppm): 156.25, 152.89, 144.34, 143.38, 141.71, 141.61, 141.20, 140.40, 140.25, 140.03, 139.57, 138.60, 136.13, 135.54, 133.78, 128.77, 128.36, 128.27, 128.07, 126.02, 122.67, 120.28, 117.63, 116.02, 115.83, 115.63, 111.48, 62.83, 62.40, 48.23, 35.58, 35.53, 31.90, 31.70, 31.29, 31.27, 30.35, 29.32, 29.28, 29.18, 26.84, 22.66, 22.59, 14.12, 14.10. HRMS (ESI) m/z calcd for $C_{78}H_{91}NS_4^+$ $(M)^+$ 1169.60289, found 1169.60217.

Synthesis of ***compound 8***: To a dry 100 mL two-necked round bottom flask, 10 ml anhydrous *N*, *N*-dimethylformamide (DMF) was added, and the solution was cooled to 0°C and stirred when 2 ml phosphorous oxychloride ($POCl_3$) was added by syringe under argon protection. The mixture was stirred at 0°C for 1 hour, and then compound **7** (400 mg, 0.34 mmol) in dry 1, 2-dichloroethane (20 ml) was added. Then, the mixture solution was allowed to reflux overnight. After cooling to room temperature, 100 ml water was added and the mixture was extracted with dichloromethane (DCM), and the organic layer was collected, washed with water and dried with anhydrous Na_2SO_4. After removal of the solvent under reduced pressure, the residue was purified by column chromatography on silica gel using a mixture solvent as eluent (petroleum ether/dichloromethane, v/v = 1/1) to give an orange solid (444 mg, 82%). ^{1}H NMR (400 MHz, $CDCl_3$), δ (ppm): 9.86 (s, 1H), 9.84 (s, 1H), 7.91 (s, 1H), 7.50-7.51 (m, 3H), 7.32 (d, *J* = 8.0 Hz, 4H), 7.08-7.14 (m, 12H), 3.76 (t, *J* = 7.6 Hz, 2H), 2.53-2.59 (m, 8H), 1.59 (br, 10H), 1.28-1.29 (m, 34H), 0.85-0.90 (m, 15H). ^{13}C NMR (100 MHz, $CDCl_3$), δ (ppm): 182.86, 182.76, 157.16, 154.32, 149.89, 145.49, 144.03, 143.98, 143.71, 142.29, 142.25, 141.33, 139.91, 139.37, 137.78, 136.87, 129.90, 128.70, 128.63, 128.55, 127.88, 124.59, 118.40, 116.92, 116.56, 63.00, 62.58, 53.48, 48.37, 35.60, 35.55, 31.90, 31.72, 31.30, 30.47, 29.34, 29.29, 29.20, 29.18, 16.87, 22.68, 22.62, 22.61, 14.13. HRMS (ESI) m/z calcd for $C_{80}H_{92}NO_2S_4^+$ $(M+H)^+$ 1226.60054, found 1226.60022.

Synthesis of ***N8IT***: To a 100 ml round bottom flask, compound **8** (150 mg, 0.122 mmol) and IC-Cl (111 mg, 0.49 mmol) were added under argon protection. Then, deoxidized chloroform (40 ml) was added and stirred for a while when pyridine (1 ml) was added. The mixture was kept stirring at 75°C for 12 h. After removal of chloroform of reaction mixture under reduced pressure, 100 ml methanol was added and the precipitate was collected by filtration. The residue was purified by column chromatography on silica gel using a mixture solvent as eluent (petroleum ether/dichloromethane, v/v = 1/1) to give a dark solid (171 mg, 85%). ^{1}H NMR (400 MHz, $CDCl_3$), δ (ppm): 8.85 (s, 1H), 8.83 (s, 1H), 8.51-8.61 (m, 2H), 7.65-7.82 (m, 4H), 7.56 (s, 2H), 7.33 (d, *J* = 7.6 Hz, 4H), 7.15-7.21 (m, 12H), 3.76 (m, 2H), 2.57-2.59 (m, 10H), 1.59 (m, 8H), 1.29 (m, 34H), 0.87 (m, 15H). ^{13}C NMR (100 MHz, $CDCl_3$), δ (ppm): 187.08, 186.94, 159.30, 158.90, 158.12, 156.78, 155.57, 153.93, 152.65, 150.85, 148.87, 147.85, 147.38, 147.24, 147.05, 146.53, 143.61, 142.68, 142.53, 142.35, 141.76, 141.27, 140.98, 140.53, 139.89, 139.46, 139.30, 138.94, 138.63, 138.35, 138.02, 137.73, 137.30, 137.18, 136.98, 136.42, 135.71, 134.99, 134.51, 134.17, 133.88, 132.82, 128.88, 128.74, 128.52, 128.27, 127.89, 127.50, 126.06, 125.58, 125.32, 125.00, 124.82,

124.68, 123.30, 121.19, 120.97, 119.83, 119.61, 117.99, 117.60, 117.27, 116.81, 115.30, 114.50, 63.14, 62.68, 48.53, 35.63, 35.56, 31.91, 31.71, 31.31, 31.28, 30.46, 29.39, 29.29, 29.21, 26.83, 22.70, 22.62, 14.13. MALDI-TOF-MS m/z: [M] calcd. for $C_{104}H_{97}Cl_2N_5O_2S_4$, 1645.59, found 1645.76.

*Synthesis of **a-IT***: To a 100 ml round bottom flask, compound **9** (100 mg, 0.093 mmol) and IC-Cl (85 mg, 0.37 mmol) were added under argon protection. Then, deoxidized chloroform (30 ml) was added and stirred for a while when pyridine (1 ml) was added. The mixture was kept stirring at 75°C for 12 h. After removal of chloroform of reaction mixture under reduced pressure, 100 ml methanol was added and the precipitate was collected by filtration. The residue was purified by column chromatography on silica gel using a mixture solvent as eluent (petroleum ether/dichloromethane, v/v = 1/1) to give a dark solid (122 mg, 88%). ^{1}H NMR (400 MHz, $CDCl_3$), δ (ppm): 8.89-8.92 (m, 2H), 8.53-8.64 (m, 2H), 7.77-7.96 (m, 4H), 7.56-7.71 (m, 4H), 7.17 (q, J_1 = 8.4 Hz, 16 H), 2.56-2.61 (m, 8H), 1.58-1.60 (m, 8H), 1.26-1.32 (m, 24H), 0.84-0.89 (m, 12H). ^{13}C NMR (100 MHz, $CDCl_3$), δ (ppm): 187.20, 187.06, 160.39, 159.30, 158.97, 158.64, 157.78, 156.66, 154.99, 149.06, 148.22, 146.23, 143.11, 143.05, 142.64, 142.39, 141.92, 141.22, 141.17, 140.97, 140.31, 138.76, 138.70, 138.33, 138.29, 137.95, 135.26, 134.90, 133.91, 128.85, 128.77, 127.79, 127.74, 126.46, 125.33, 124.73, 123.81, 122.02, 121.37, 119.73, 118.30, 114.61, 114.40, 114.28, 114.15, 70.21, 69.00, 63.23, 62.96, 35.57, 31.71, 31.69, 31.35, 31.28, 29.11, 22.60, 22.58, 14.12, 14.10. MALDI-TOF-MS m/z: [M] calcd. for $C_{94}H_{80}Cl_2N_4O_2S_4$, 1494.45, found 1494, 22.

Measurements

^{1}H NMR and ^{13}C NMR spectra were recorded on a Bruker Advanced II (400 MHz) spectrometer. The high-resolution mass spectra (HRMS) and matrix-assisted laser desorption/ionization time of flight mass spectrometry (MALDI-TOF-MS) were performed on Thermo Scientific LTQ Orbitrap Xl using ESI and 5800 MALDI-TOF/TOF mass spectrometry (AB SCIEX, USA) in positive mode, respectively. UV-vis spectra were measured using a Shimadzu UV-2500 recording spectrophotometer. Cyclic voltammetry (CV) measurements of targeted SMA thin films were conducted on a CHI voltammetric analyzer in acetonitrile solution with 0.1 M tetrabutylammonium hexafluorophosphate (n-Bu_4NPF_6) as supporting electrolyte at room temperature by using a scan rate of 100 mV s^{-1} and conventional three-electrode configuration. Density functional theory (DFT) calculations were performed at the B3LYP/6-31G(d,p) level. Atomic force microscopy (AFM) images were

obtained by using a NanoMan VS microscope in the tapping-model. GIWAXS measurements were performed in Chinese University of Hong Kong.

Devices fabrication and characterization

Organic solar cells (OSCs) were fabricated with a device structure of ITO/PEDOT: PSS/active layer/ZrAcac/Al. The ITO-coated glass was washed by detergent and then cleaned inside an ultrasonic bath by using deionized water, acetone, and isopropyl alcohol sequentially and dried overnight in an oven. Before use, the glass substrates were treated in a UV-Ozone Cleaner for 20 min to improve its work function and clearance. A thin PEDOT: PSS (Heraeus Clevios P VP A 4083) layer with a thickness of about 40 nm was spin-cast onto the ITO substrates at 4000 rmp for 40 s, and then dried at 150 °C for 10 min in air. The PEDOT:PSS coated ITO substrates were fast transferred to a N_2 filled glove-box for further processing. The donor:acceptor blends with weight ratio of 1:1 and total concentration of 16 mg/mL were dissolved in chloroform and 0.25% DIO was added. Then the solution was stirred overnight for intensive mixing in a nitrogen-filled glove box. The blend solution was spin-cast on the top of PEDOT: PSS layer at 2700 rmp for 40 s. Then it was annealed at 100 °C for 5 min to remove the residual solvent. Subsequently, the active layer coated substrates were quickly transferred to a glove-box integrated thermal evaporator for electrode deposition. A thin Zracac layer and Al layer (100 nm) were sequentially evaporated under vacuum of 5×10^{-5} Pa through a shadow mask. The active area of each device was 5.90 mm^2 controlled by a shadow mask. The optimal blend thickness measured on a Bruker Dektak XT stylus profilometer was about 110 nm. The current-voltage (*J-V*) characteristic curves of all packaged devices were measured by using a Keithley 2400 Source Meter in air. Photocurrent was measured in an Air Mass 1.5 Global (AM 1.5 G) solar simulator (Class AAA solar simulator, Model 94063A, Oriel) with an irradiation intensity of 100 mW cm^{-2}, which was measured by a calibrated silicon solar cell and a readout meter (Model 91150V, Newport). IPCE spectra were measured by using a QEX10 Solar Cell IPCE measurement system (PV measurements, Inc.).

SCLC

The electron and hole mobility of N7ITand N8IT neat and blend films were measured by using the method of space-charge limited current (SCLC). The electron-only SCLC device was a stack of ITO/ZnO/active layer/ZrAcac/Al, and the hole-only device was a stack of ITO/MoO_x/active layer/ MoO_x /Al. The electron-only and hole-only SCLC devices fabricating methods were same with those for solar cells. The charge carrier mobility was determined by

fitting the dark current to the model of a single carrier SCLC according to the equation: $J = 9\varepsilon_0\varepsilon_r\mu V^2/8d^3$, where J is the current density, d is the film thickness of the active layer, μ is the charge carrier mobility, ε_r is the relative dielectric constant of the transport medium, and ε_0 is the permittivity of free space. $V = V_{app} - V_{bi}$, where V_{app} is the applied voltage, V_{bi} is the offset voltage. The carrier mobility can be calculated from the slope of the $J^{1/2} \sim V$ curves.

TPV and CE measurements

A 405 nm laser diode was used to keep the devices in approximately V_{oc} conditions. Driving the laser intensity with a waveform generator (Agilent 33500B) and measuring the light intensity with a highly linear photodiode allowed reproducible adjustments of the light intensity with an error below 0.5% over a range from 0.15 to 2.5 suns. A small perturbation was induced with a second 405 nm laser diode driven by a function generator from Agilent. The intensity of the short (60 ns) laser pulse was adjusted to keep the voltage perturbation below 10 mV, typically at 5 mV. After the pulse, the voltage decays back to its steady state value in single exponential decay. The characteristic decay time was determined from a linear fit to a logarithmic plot of the voltage transient, and returned the small perturbation charge carrier lifetime. In CE measurements, a 405 nm laser diode illuminated the device for 200 μs, which was sufficient to reach a constant V_{oc} with steady state conditions. At the end of the illumination period, an analogue switch was triggered that switched the solar cell from open-circuit to short-circuit (50 U) conditions within less than 50 ns.

TPC measurements

OSCs were excited with a 405 nm laser diode. The transient photocurrent response of the devices at short circuit condition to a 200 μs square pulse from the LED with no background illumination. The current traces were recorded on an ektronix DPO3034 digital oscilloscope by measuring the voltage drop over a 5 ohm sensor resistor in series with the solar cell. DC voltage was applied to the solar cell with an MRF544 bipolar junction transistor in common collector amplifier configuration.

FTPS-EQE measurements

FTPS-EQE spectra were measured by using a Vertex 70 from Bruker optics and QTH lamp. The EL signature was collected with monochromator and detected with Si-CCD detector.

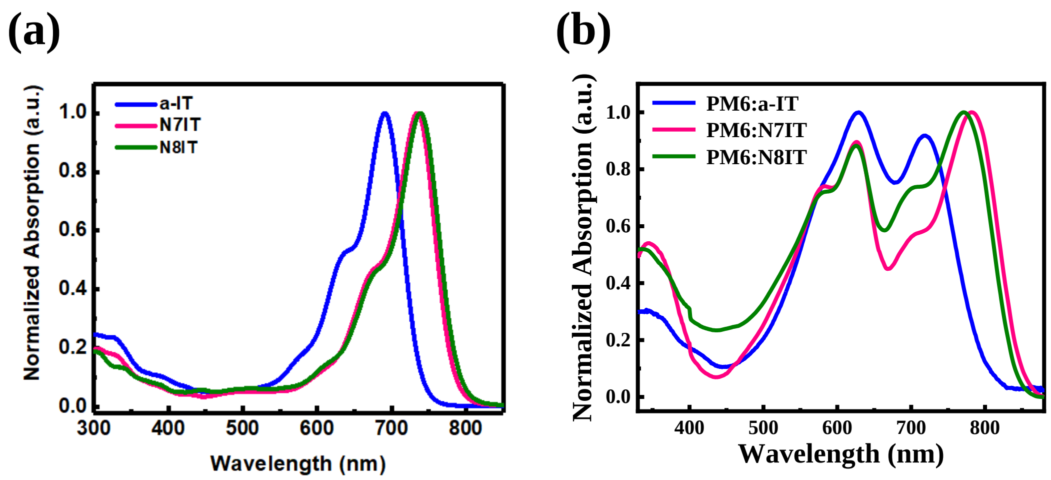

Figure S1. (a) Normalized UV-vis absorption spactra of a-IT, N7IT and N8IT in solution. (b) Normalized UV-vis absorption spactra of PM6:a-IT, PM6:N7IT and PM6:N8IT blend films.

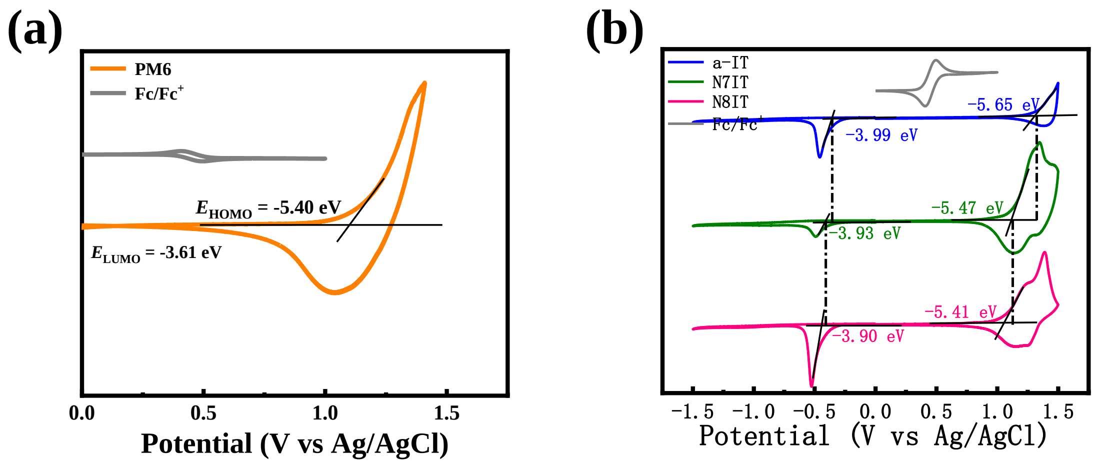

Figure S2 (a) The CV curve of PM6 (E_{LUMO} = E_{HOMO} + E_{gap}). (b) The CV curves of a-IT, N7IT and N8IT.

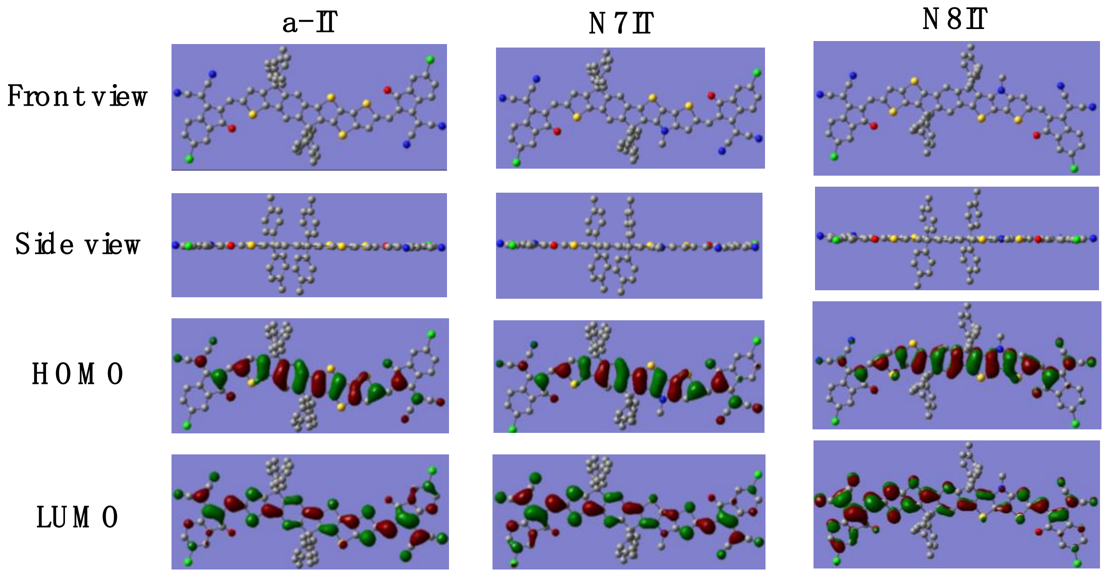

Figure S3. DFT calculation results.

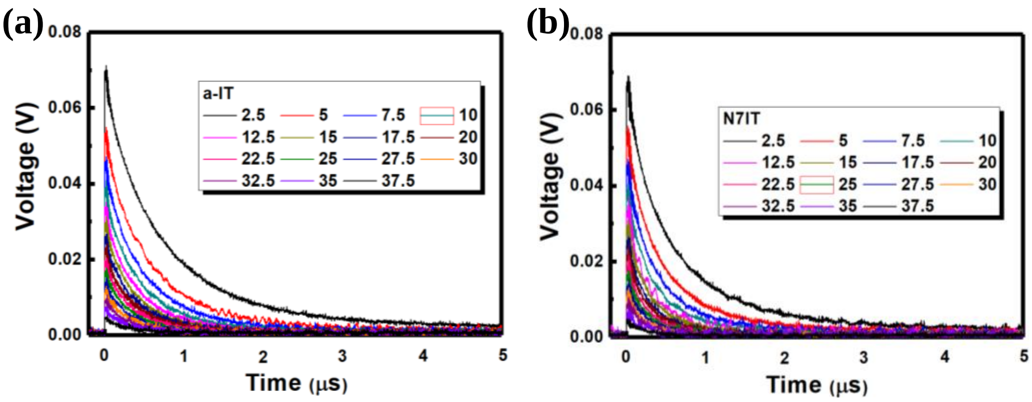

Figure S4. Transient photo-voltage (TPV) measurements of (a) a PM6:a-IT-based and (b) a PM6:N7IT-based OSCs. The strong dependence of light intensity and lifetime can be observed.

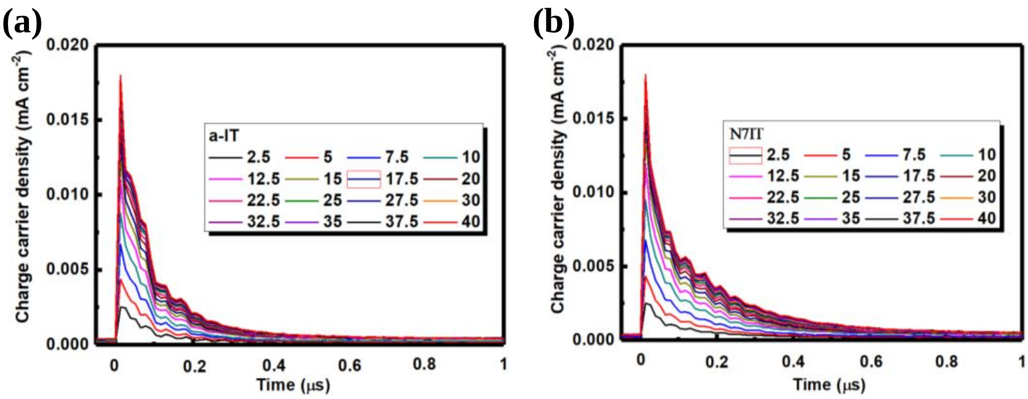

Figure S5. Charge extraction (CE) measurements of (a) a PM6:a-IT-based and (b) a PM6:N7IT-based OSCs. The strong dependence of light intensity and lifetime can be observed.

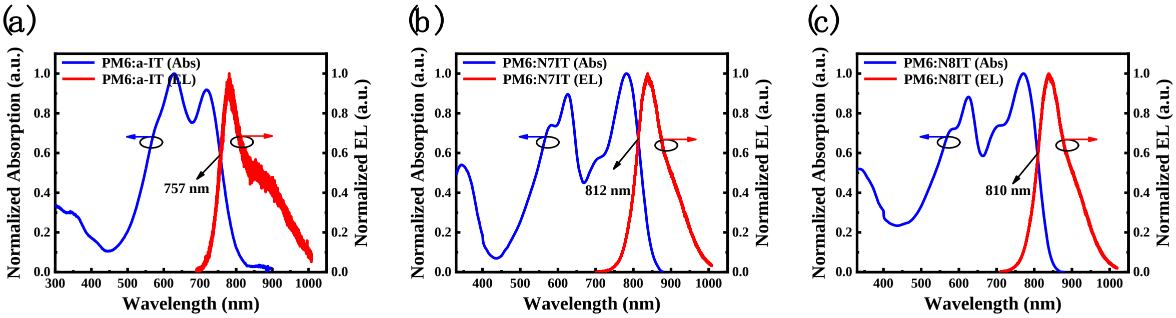

Figure S6. Normalized UV-Vis absorption and EL spectra for (a) PM6:a-IT, (b) PM6:N7IT and (c) PM6:N8IT blend films.

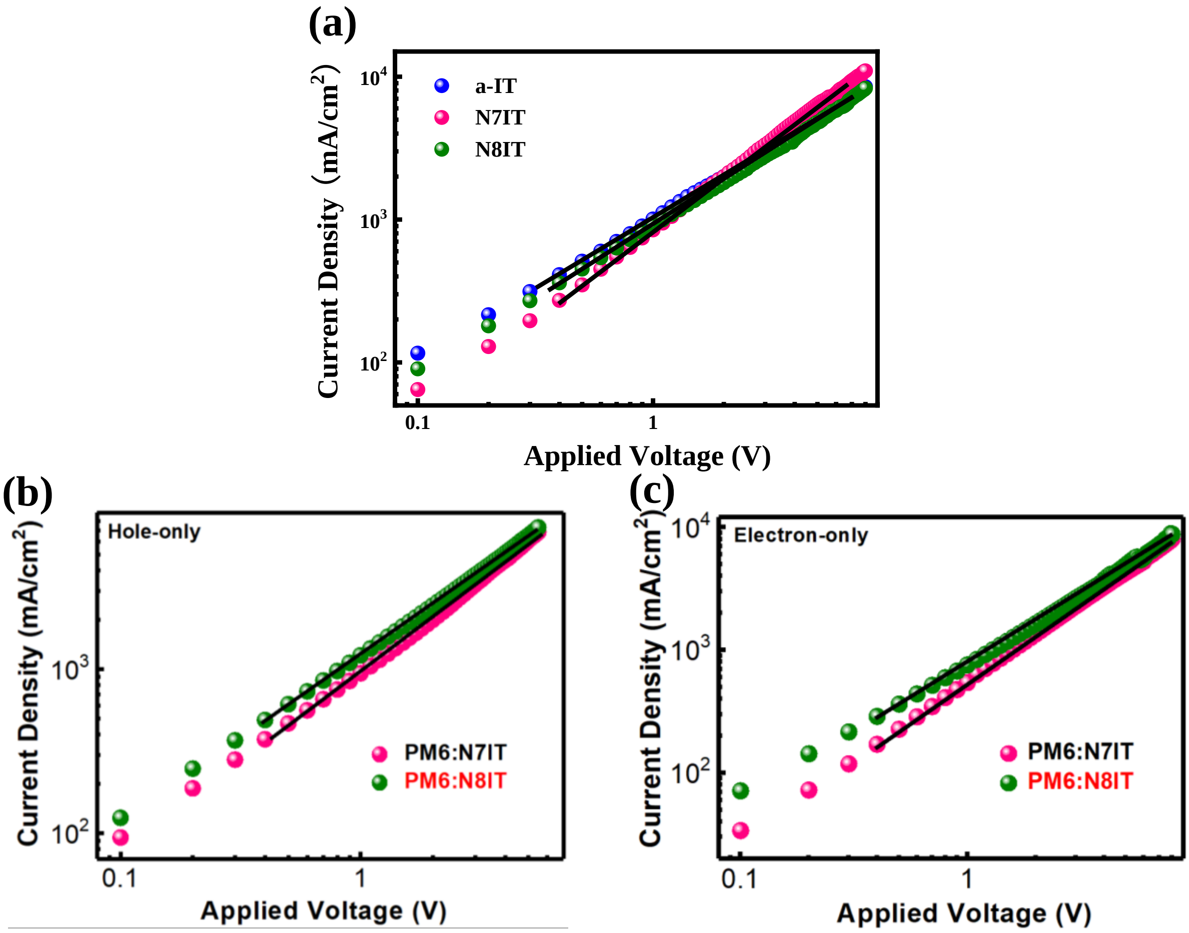

Figure S7. SCLC measurements for: (a) electron-only devices based on a-IT, N7IT and N8IT neat films; (b) hole-only devices based on PM6:N7IT and PM6:N8IT blend films; (c) electron-only devices based on PM6:N7IT and PM6:N8IT blend films.

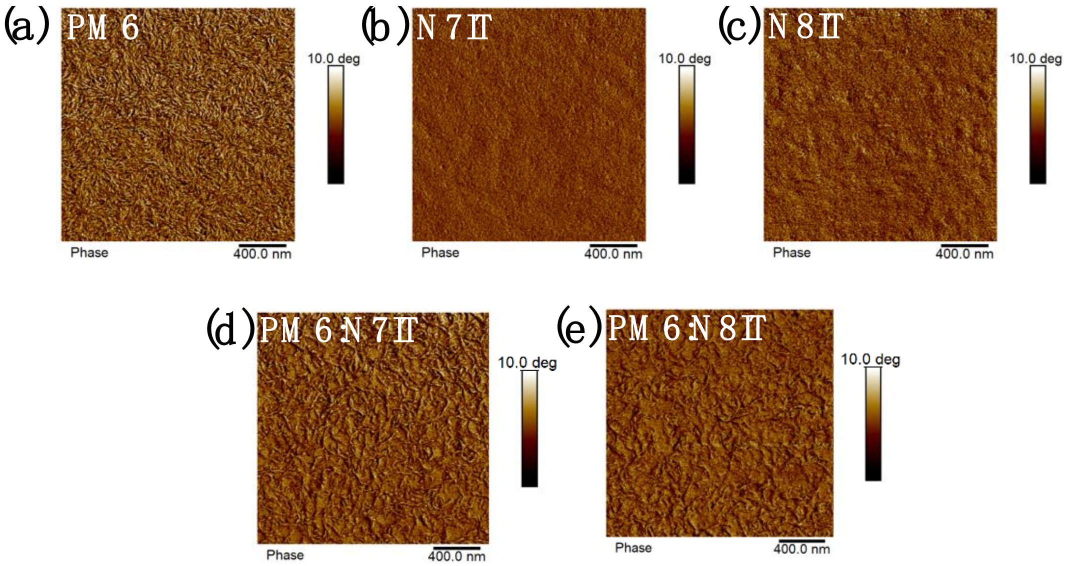

Figure S8. AFM phase images for: (a) a PM6 neat film; (b) a N7IT neat film; (c) a N8IT film; (d) a PM6:N7IT blend film; (e) a PM6:N8IT blend film.

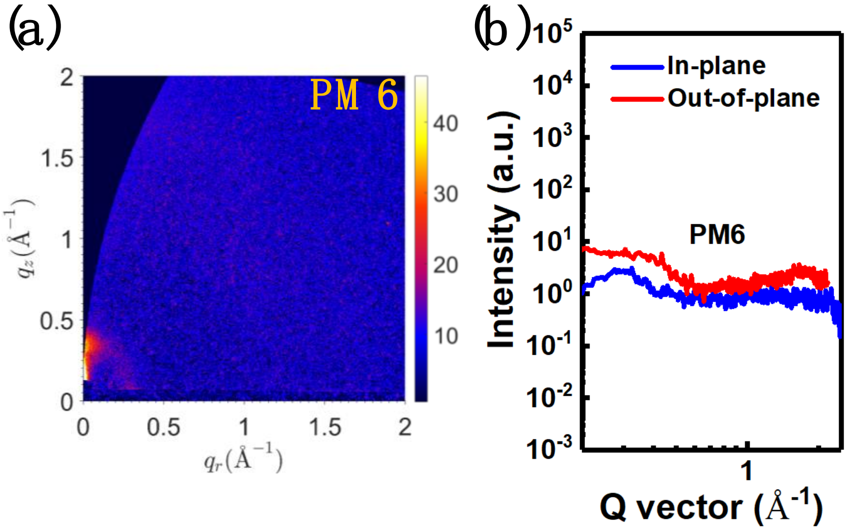

Figure S9. The GIWAXS measurements for PM6: (a) 2D-GIWAXS pattern and (b) corresponding cut-line profile.

Table S1 The E_{gap} and E_{loss} obtained from absorption and EL spectra of blend films.

Active layer	E_{gap} [eV]	V_{oc} [V]	E_{loss} [eV]
PM6:a-IT	1.638	0.907	0.731
PM6:N7IT	1.527	0.932	0.595
PM6:N8IT	1.531	0.943	0.588

Table S2 Summary of V_{OC}, J_{SC}, E_{loss} and PCE of OSCs based on different systems.

Acceptors	Donor	V_{OC} (V)	J_{SC} (mA cm^{-2})	E_{loss} [a)] (eV)	PCE (%)	Refs.
BTOIC	PBDB-T	0.862	18.60	0.531	10.96	1
IHIC	PTB7-Th	0.754	19.01	0.627	9.77	2
F6IC	PTB7-Th	0.611	18.07	0.750	7.1	3
F8IC	PTB7-Th	0.640	25.12	0.630	10.9	3
F10IC	PTB7-Th	0.732	20.83	0.518	10.2	3
4TIC	PTB7-Th	0.78	18.8	0.598	10.43	4
SN6IC-4F	PBDB-T	0.78	23.2	0.539	13.2	4
IEICO	PBDTTT-E-T	0.82	17.7	0.52	8.4	5
ATT-2	PTB7-Th	0.73	20.75	0.589	9.58	6
BT-CIC	PCE-10	0.70	22.5	0.633	11.2	7
DTPC-IC	PTB7-Th	0.76	21.92	0.454	10.21	8
CO*i*8DFIC	PTB7-Th	0.68	26.12	0.580	12.16	9
R12-4Cl	PBDB-T	0.75	18.5	0.602	9.3	10
Y6	PM6	0.83	25.3	0.502	15.7	11
Y1	PBDB-T	0.87	22.44	0.500	13.42	12

Y2	PBDB-T	0.82	23.56	0.520	13.40	12
ACS8	PTB7-Th	0.75	25.3	0.555	13.2	13
T1	PTB7-Th	0.72	20.95	0.586	9.82	14
T2	PTB7-Th	0.65	24.85	0.631	10.87	14
T3	PTB7-Th	0.61	22.00	0.660	9.43	14
T4	PTB7-Th	0.61	18.57	0.660	7.01	14
IEICO-4F	J52	0.734	21.9	0.506	9.4	15
IPIC-4F	PBDB-T	0.835	19.8	0.544	11.1	16
IPIC-4Cl	PBDB-T	0.813	22.2	0.522	13.4	16
CPDT-4F	PBDB-T	0.68	20.1	0.632	9.47	17
CPDT-4Cl	PBDB-T	0.65	21.3	0.655	9.28	17
SiOTIC-4F	PTB7-Th	0.65	21.6	0.501	9.0	18
COTIC-4F	PTB7-Th	0.56	20.3	0.518	7.4	18
CO5DFIC-OT	PTB7-Th	0.71	17.58	0.566	7.66	19
CO5DFIC-ST	PTB7-Th	0.74	20.71	0.596	9.73	19
CO*i*6IC	FTAZ	0.82	17.45	0.553	8.43	20
CO*i*6FIC	FTAZ	0.75	19.38	0.588	9.12	20
CO*i*6DFIC	FTAZ	0.67	20.98	0.642	8.25	20
T6Me	PM6	0.87	21.33	0.509	12.09	21
BDTIT-M	PBDB-T	0.903	17.56	0.588	12.12.	22
BDTThIT-M	PBDB-T	0.942	18.03	0.647	11.31	22
NITI	PBDB-T	0.86	20.67	0.63	12.74	23
IT-M	PBDBT	0.94	17.44	0.66	12.05	24
IT-OM-2	PBDB-T	0.93	17.53	0.66	11.9	25
ITCC	PBDB-T	1.01	15.9	0.66	11.4	26
ITIC	PBDB-T	0.90	16.81	0.67	11.2	27
FTIC-C6C8	PBDB-T	0.93	18.55	0.70	11.12	28
NFBDT	PBDB-T	0.868	17.85	0.692	10.42	29
FDICTF	PBDB-T	0.94	15.81	0.69	10.06	30
IDT-BOC6	PBDB-T	1.01	17.52	0.62	9.6	31
ZITI	PBDB-T	0.89	19.80	0.64	13.03	32
IXIC-2Cl	PBDB-T	0.73	23.6	0.57	12.2	33
IXIC-4Cl	PBDB-T	0.69	22.9	0.56	11.2	33
IT-4F	PBDB-T-SF	0.88	20.5	0.719	13.10	34
ITIC-Th1	FTAZ	0.85	19.33	0.737	12.10	35
IT-M	PB3T	1.00	18.9	0.630	11.9	36
ITCPTC	PBT1-EH	0.95	16.5	0.751	11.8	37
m-ITIC	J61	0.912	18.31	0.705	11.77	38
m-ITIC	J91	0.984	18.03	0.655	11.63	39
INIC3	FTAZ	0.852	19.44	0.674	11.5	40
ITIC	PBQ-4F:	0.95	18.20	0.668	11.34	41
BT-IC	J71	0.90	17.75	0.657	10.46	42
IEICO-4F	PBDTTT-EFT	0.739	22.5	0.594	10.0	43

[a]Calculated from $E_{loss} = E_g^{opt} - eV_{OC}$.

Table S3 Key photovoltaic parameters calculated from the J_{ph}-V_{eff} curves of PM6:a-IT, PM6:N7IT- and PM6:N8IT-based OSCs.

Active layer	J_{sat}[a] (mA cm^{-2})	J_{ph}^{*b} (mA cm^{-2})	$J_{ph}^{\&c}$ (mA cm^{-2})	J_{ph}^{*}/J_{sat} (%)	$J_{ph}^{\&}/J_{sat}$ (%)
PM6:a-IT	17.20	16.60	14.88	96.5	86.5
PM6:N7IT	22.52	21.04	18.10	93.4	80.4
PM6:N8IT	19.90	18.53	14.89	93.1	74.8

[a]The J_{ph} under condition of V_{eff} = 2.0 V, [b]The J_{ph} under short circuit condition. [c]The J_{ph} under maximum power output condition.

Table S4 Morphological parameters obtained from GIWAXS cut-line profiles in out-of-plane direction.

Samples	(100)				(010)			
	location (Å^{-1})	*d*-spacing (Å)	FWHM (Å^{-1})	CL (Å)	location (Å^{-1})	*d*-spacing (Å)	FWHM (Å^{-1})	CL (Å)
PM6	-	-	-	-	1.72	3.65	1.23	4.60
N7IT	-	-	-	-	1.75	3.58	0.30	21.03
N8IT	0.27	22.7	0.04	151.1	1.72	3.64	0.32	17.9
PM6:N7IT	0.28	22.5	0.05	124.7	1.73	3.62	0.35	16.4
PM6:N8IT	0.28	22.7	0.04	142.1	1.71	3.68	0.48	11.7

Table S5 Morphological parameters obtained from GIWAXS cut-line profiles in in-plane direction.

Samples	(100)				(010)			
	location (Å^{-1})	*d*-spacing (Å)	FWHM (Å^{-1})	CL (Å)	location (Å^{-1})	*d*-spacing (Å)	FWHM (Å^{-1})	CL (Å)
PM6	0.29	21.4	0.12	47.2	-	-	-	-
N7IT	0.32	19.9	0.11	48.8	-	-	-	-
N8IT	0.29	21.6	0.06	92.9	-	-	-	-
PM6:N7IT	0.29	21.7	0.07	80.3	-	-	-	-
PM6:N8IT	0.29	21.6	0.06	94.8	-	-	-	-

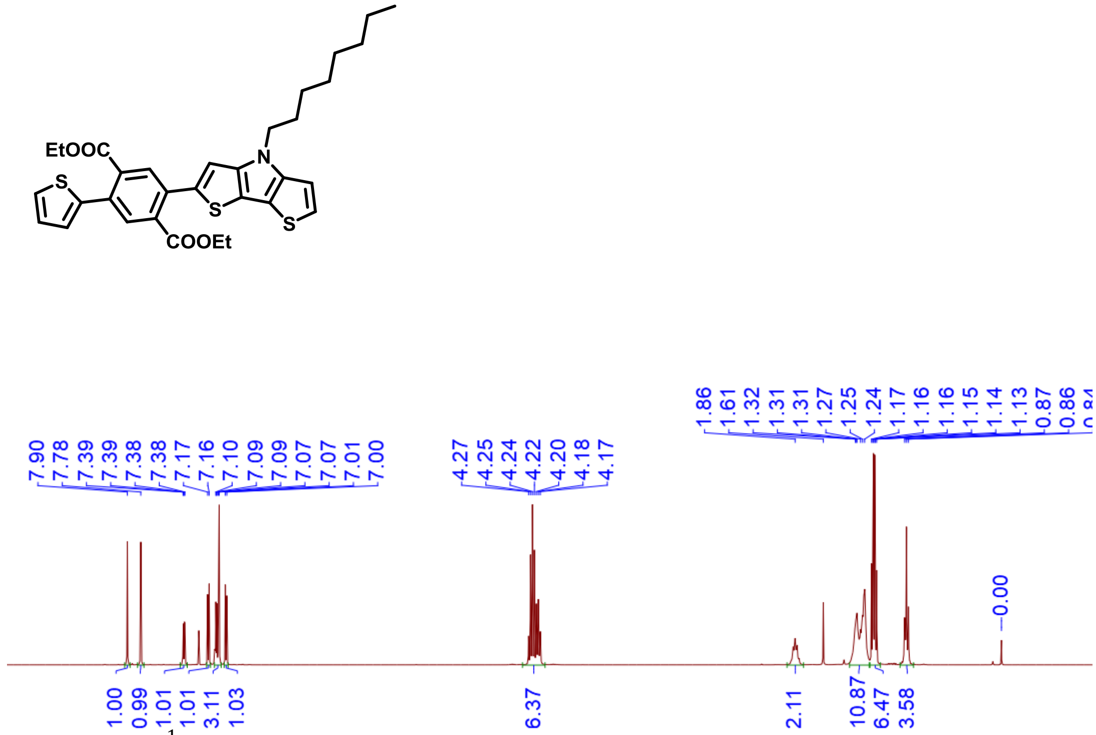

Figure S10. The ^{1}H NMR spectrum of compound **2**.

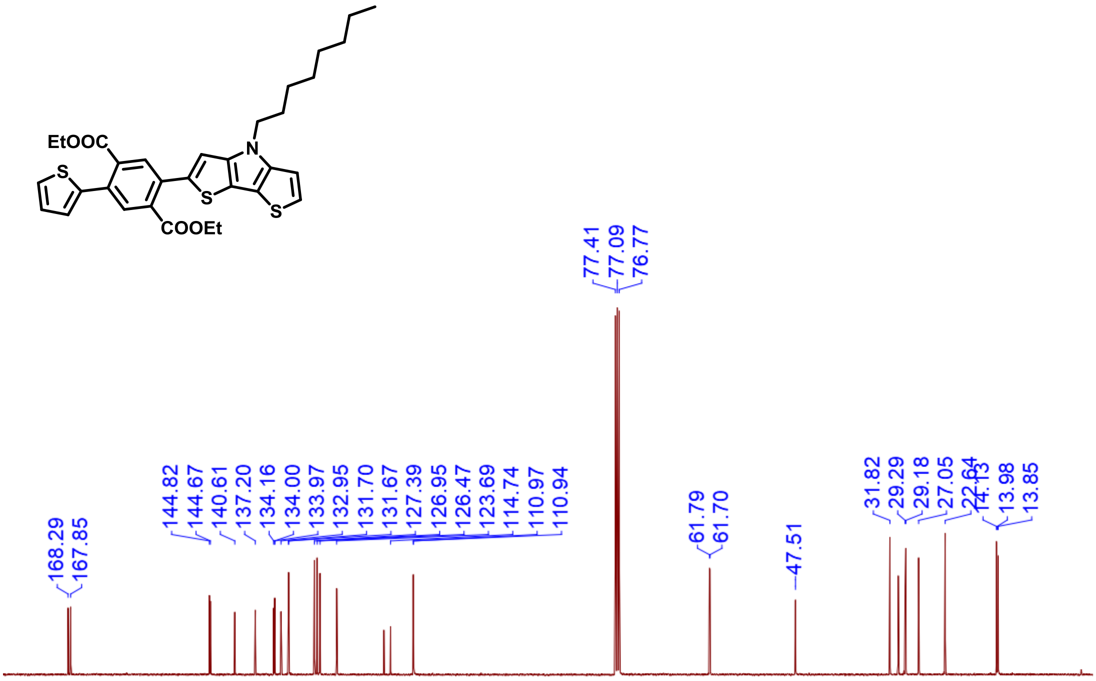

Figure S11. The ^{13}C NMR spectrum of compound **2**.

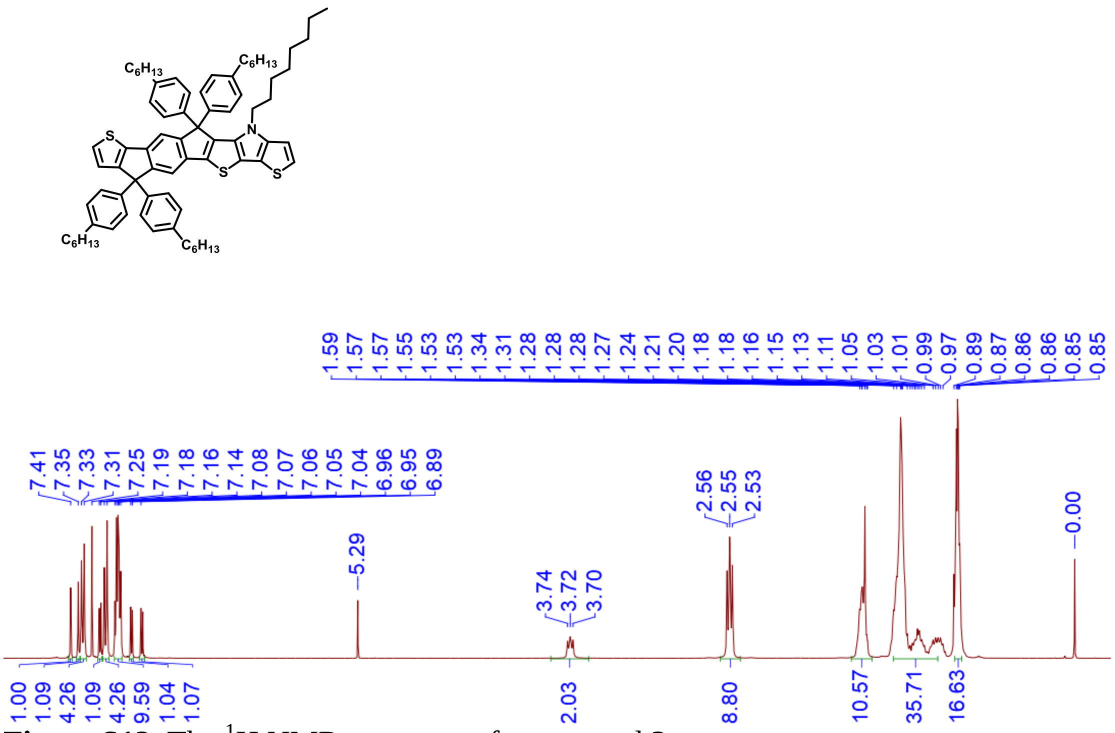

Figure S12. The ^{1}H NMR spectrum of compound **3**.

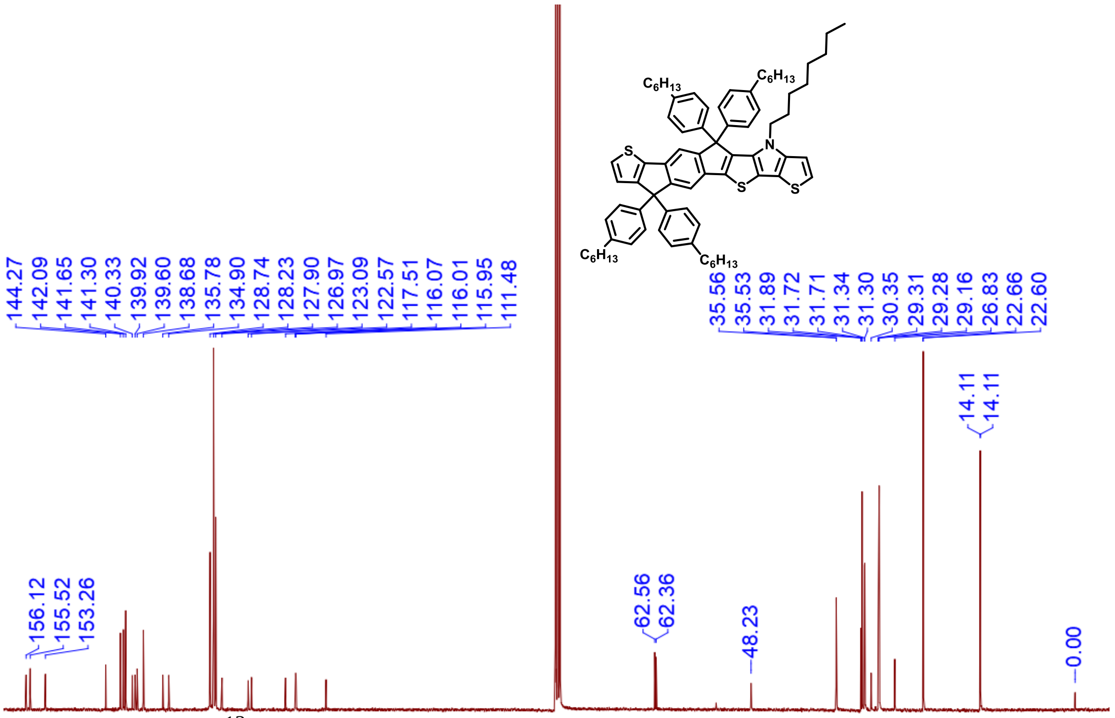

Figure S13. The ^{13}C NMR spectrum of compound **3**.

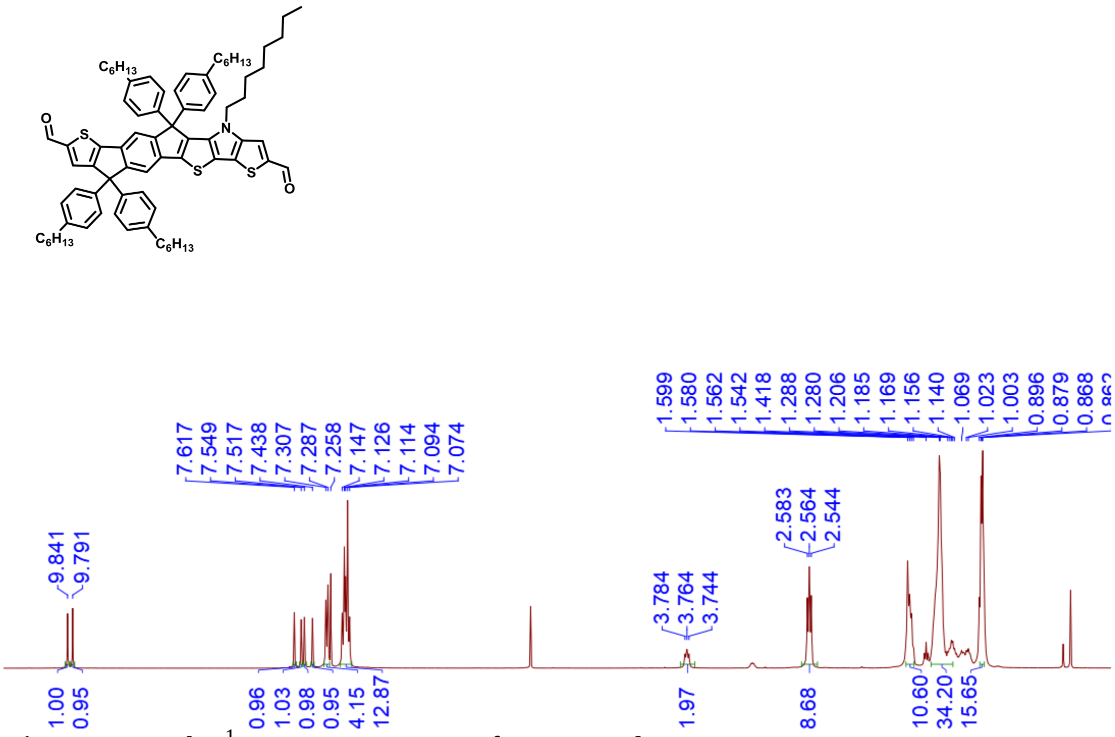

Figure S14. The ^{1}H NMR spectrum of compound **4**.

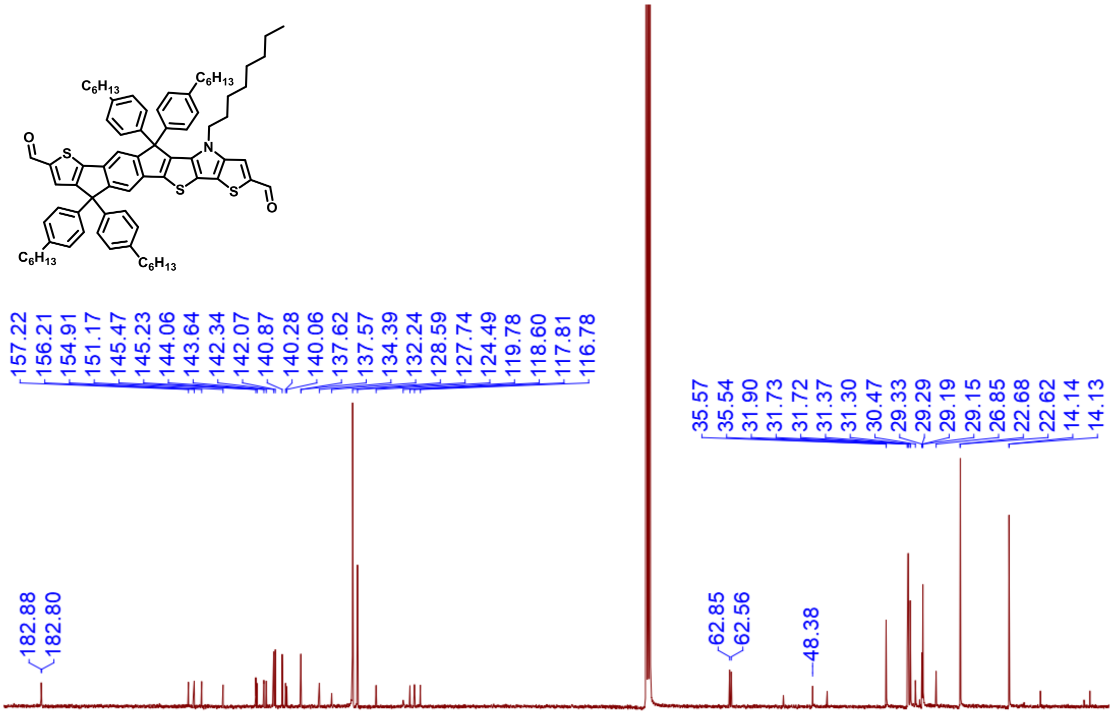

Figure S15. The ^{13}C NMR spectrum of compound **4**.

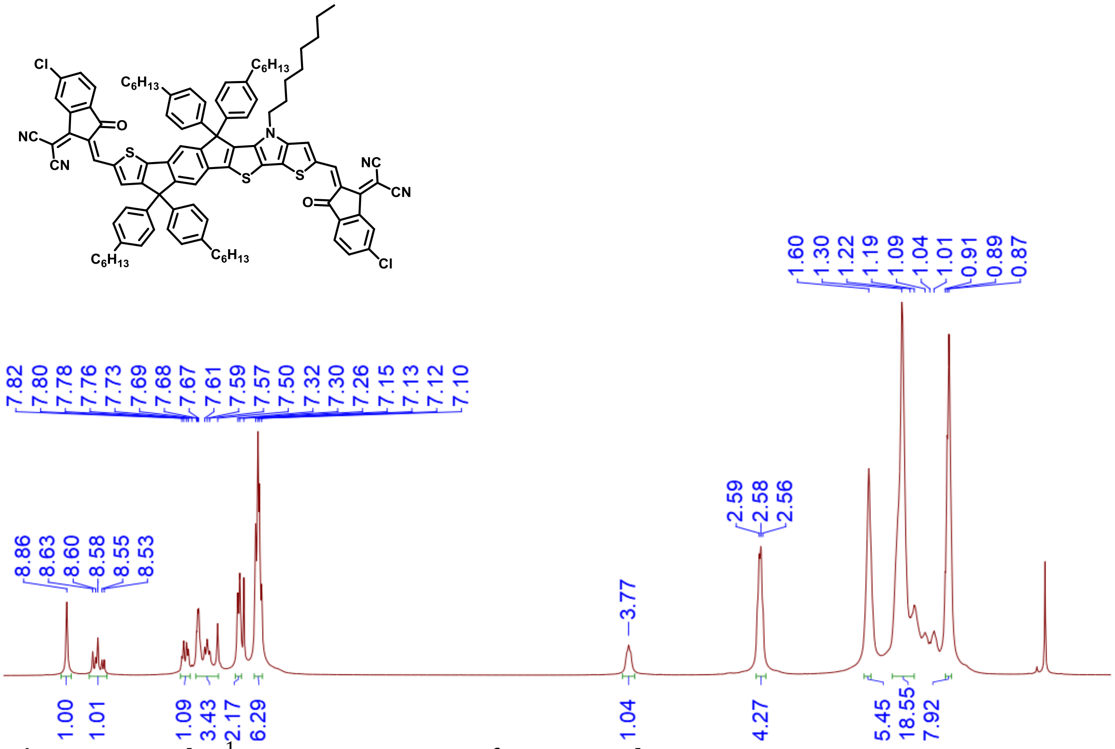

Figure S16. The ^{1}H NMR spectrum of compound **N7IT**.

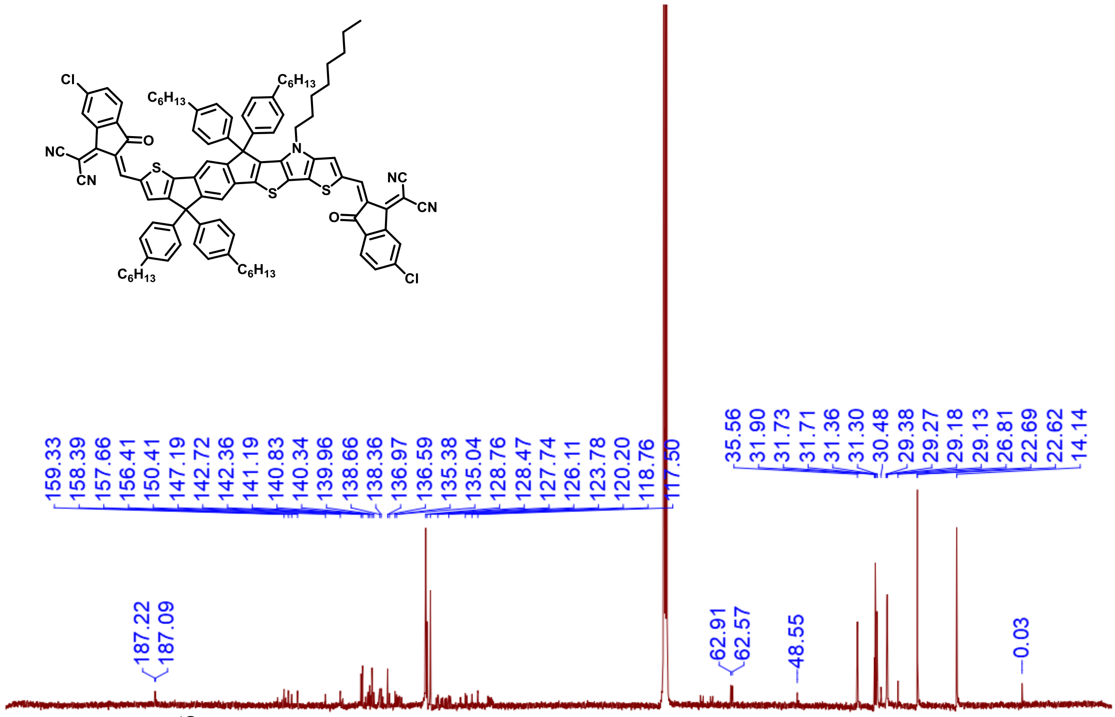

Figure S17. The ^{13}C NMR spectrum of compound **N7IT**.

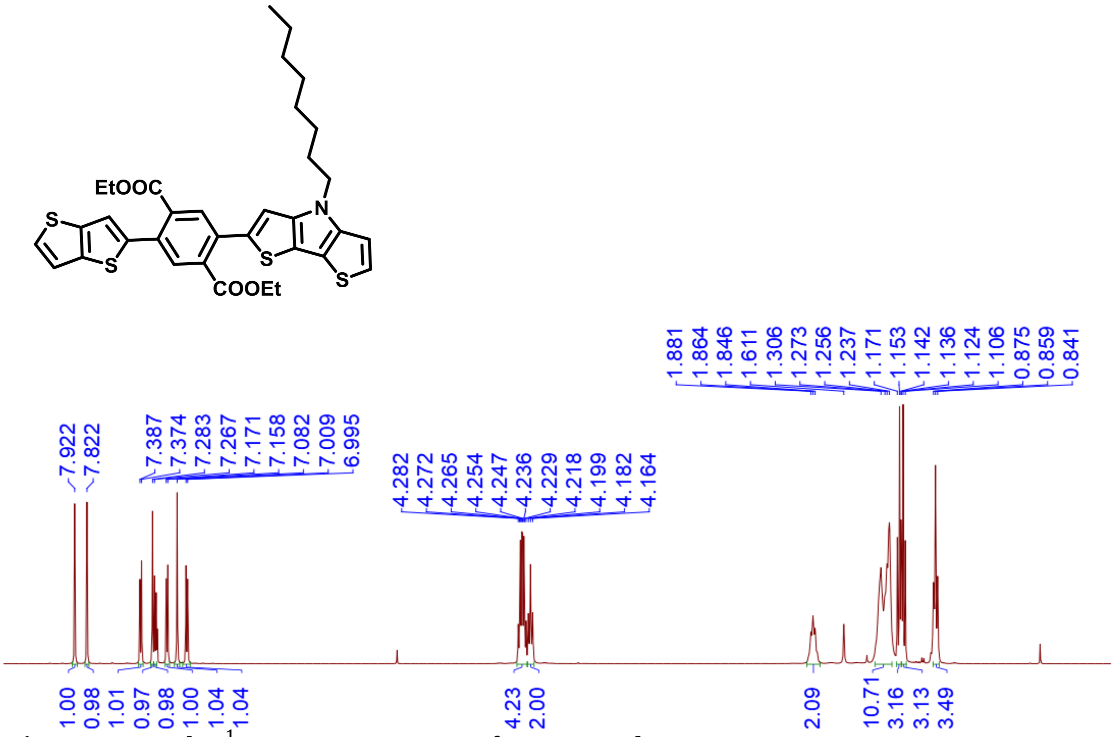

Figure S18. The ^{1}H NMR spectrum of compound **6**.

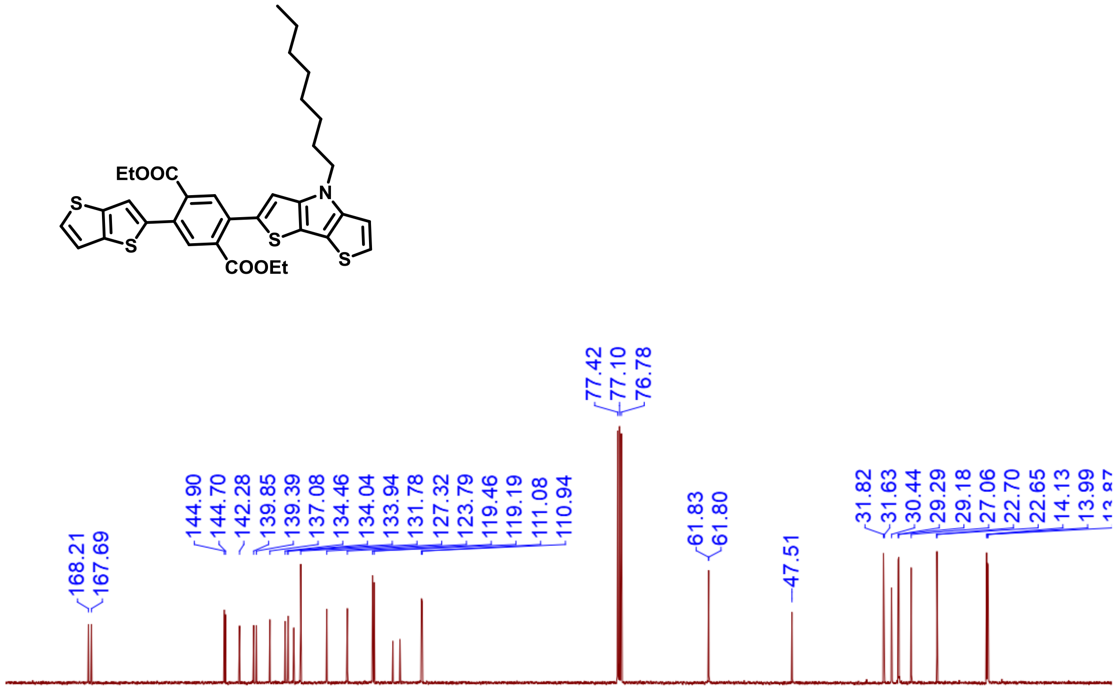

Figure S19. The ^{13}C NMR spectrum of compound **6**.

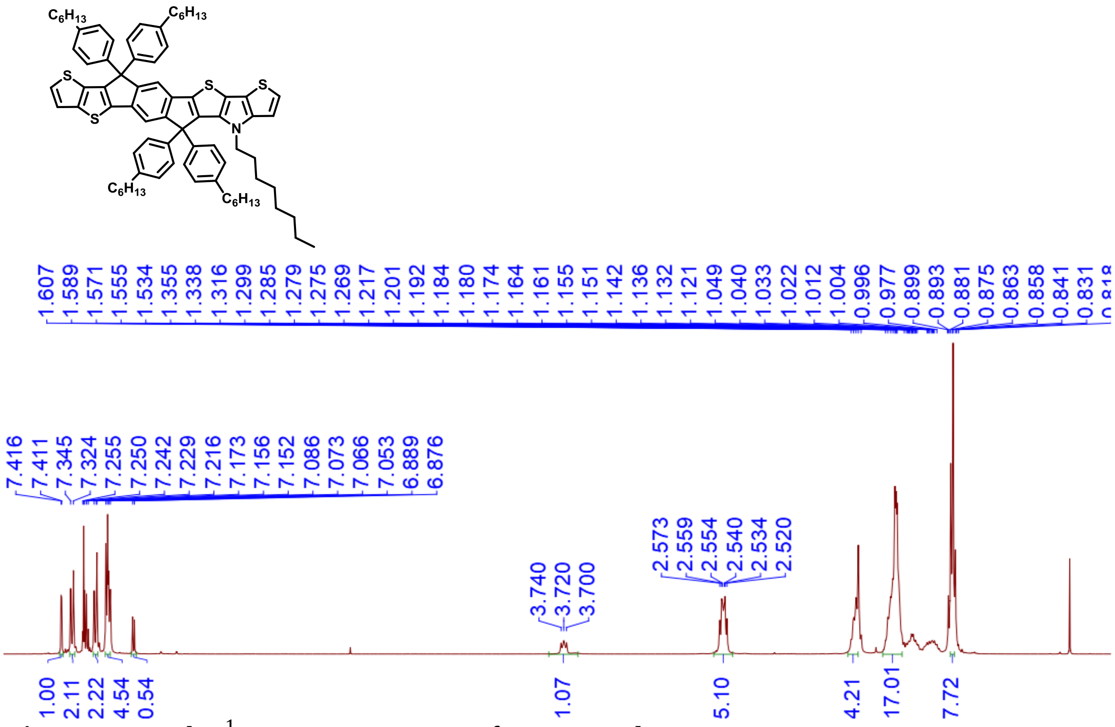

Figure S20. The ^{1}H NMR spectrum of compound **7**.

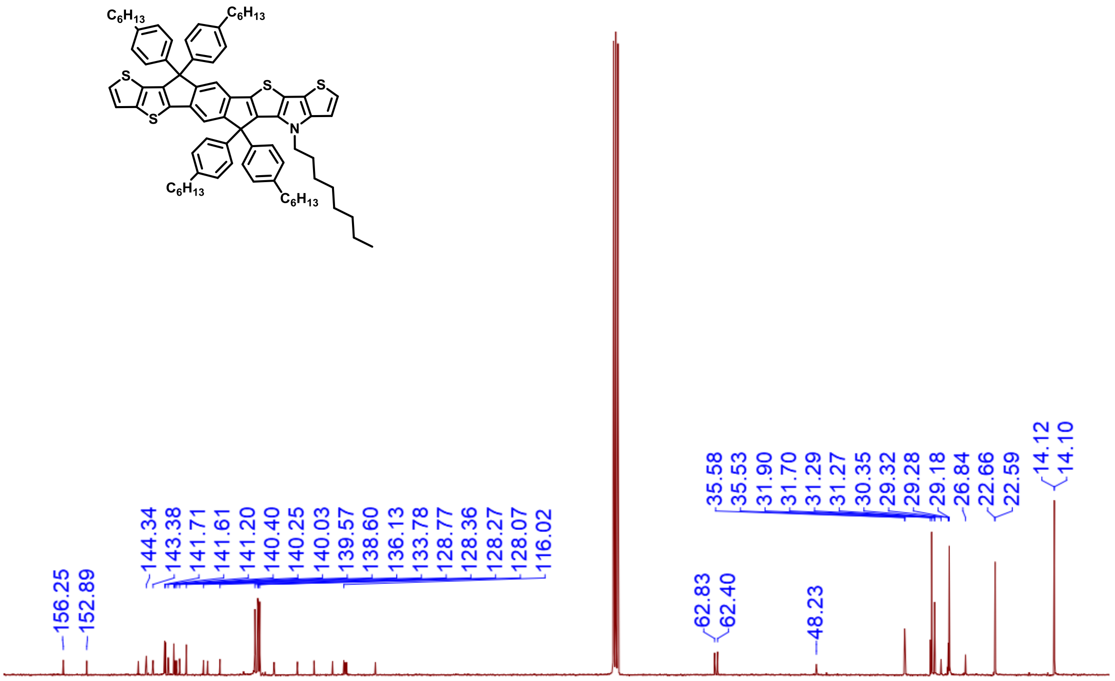

Figure S21. The ^{13}C NMR spectrum of compound **7**.

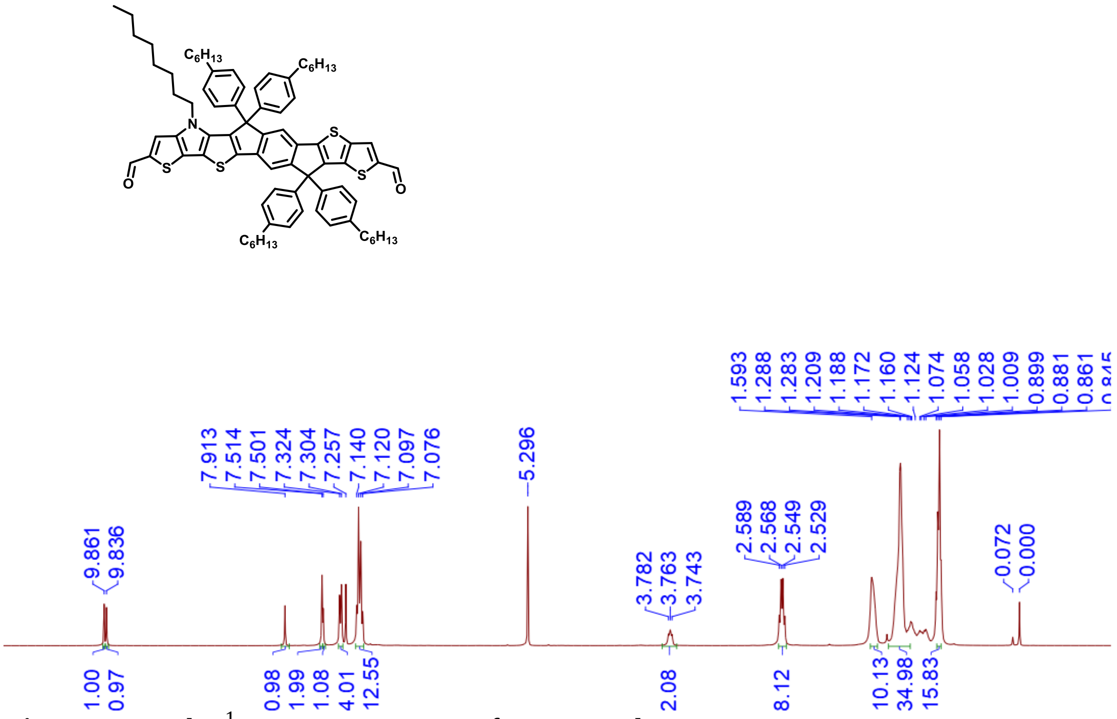

Figure S22. The ^{1}H NMR spectrum of compound **8**.

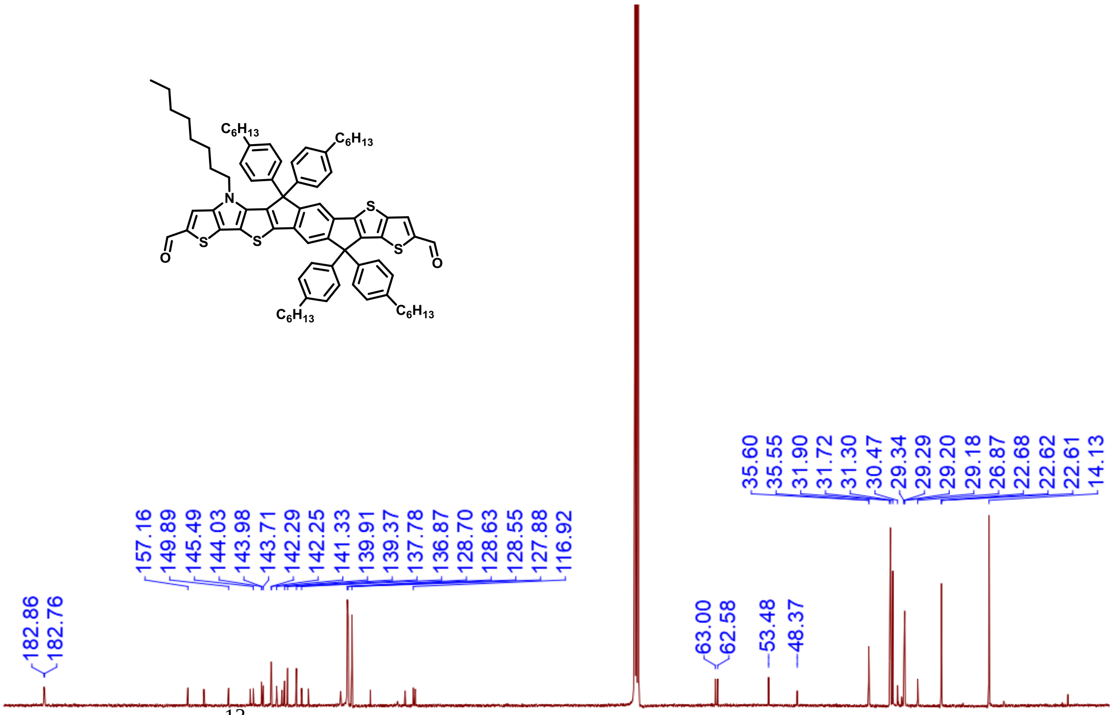

Figure S23. The ^{13}C NMR spectrum of compound **8**.

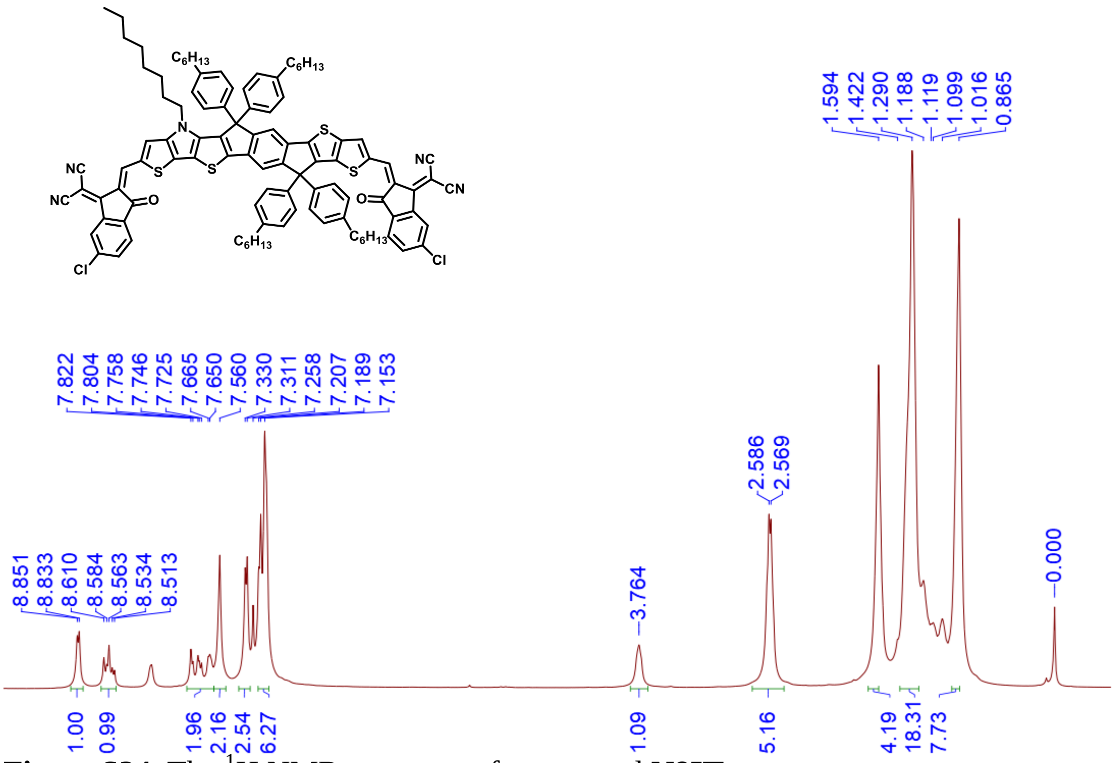

Figure S24. The ^{1}H NMR spectrum of compound **N8IT**.

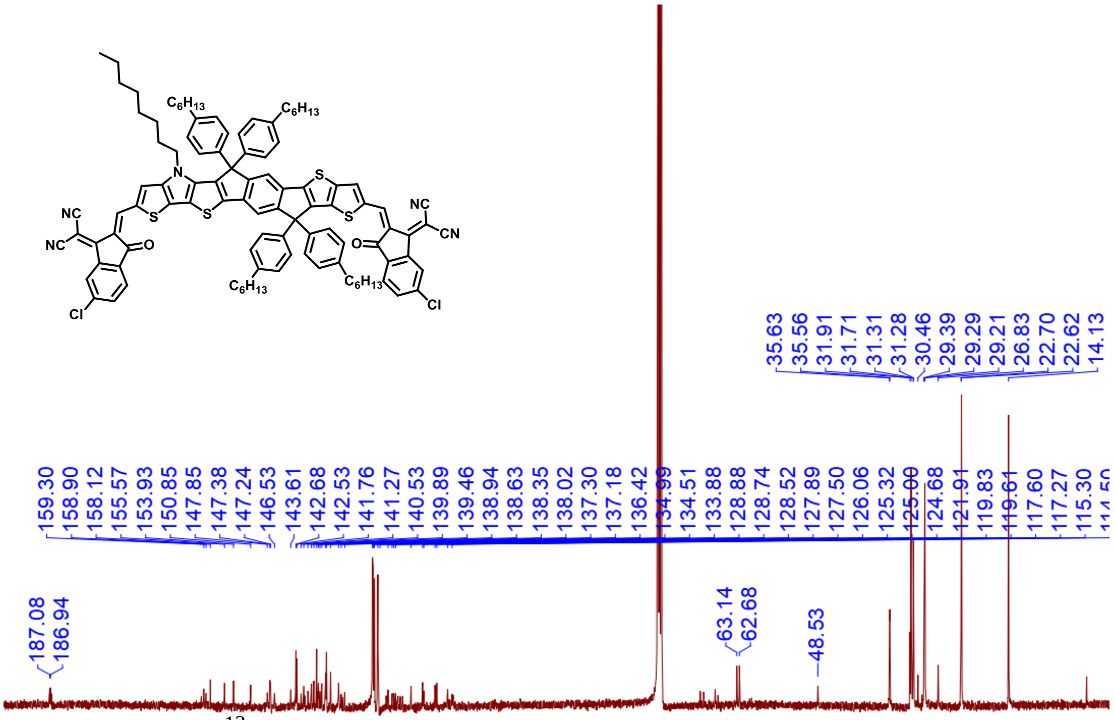

Figure S25. The ^{13}C NMR spectrum of compound **N8IT**.

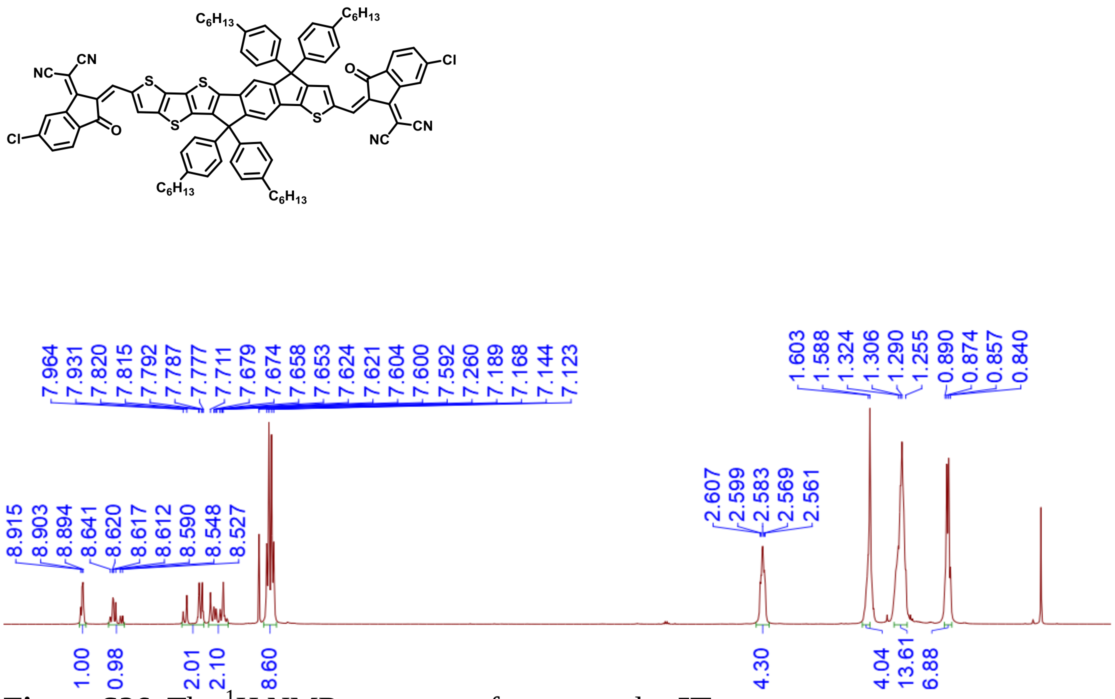

Figure S26. The ^{1}H NMR spectrum of compound **a-IT**.

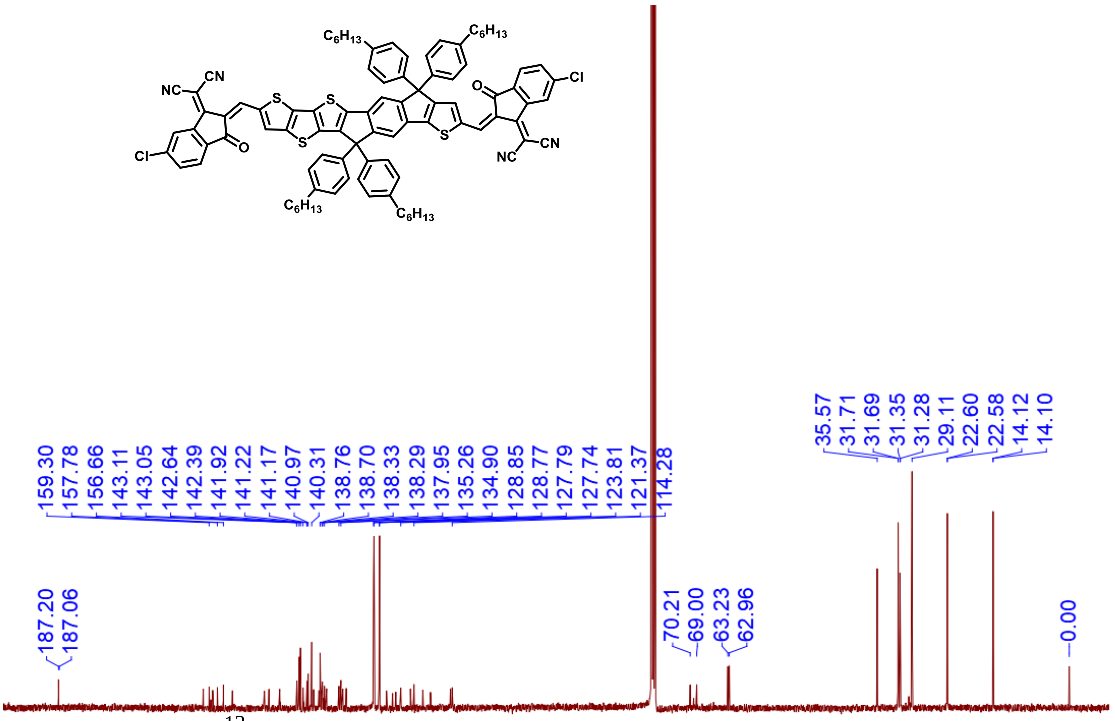

Figure S27. The ^{13}C NMR spectrum of compound **a-IT**.

References

[1] W. Gao, T. Liu, R. Ming, Z. Luo, K. Wu, L. Zhang, J. Xin, D. Xie, G. Zhang, W. Ma, H. Yan, C. Yang, *Adv. Funct. Mater.* **2018**, *28*, 1803128.

[2] W. Wang, C. Yan, T.-K. Lau, J. Wang, K. Liu, Y. Fan, X. Lu, X. Zhan, *Adv. Mater.* **2017**, *29*, 1701308.

[3] S. Dai, T. Li, W. Wang, Y. Xiao, T.-K. Lau, Z. Li, K. Liu, X. Lu, X. Zhan, *Adv. Mater.* **2018**, *30*, 1706571.

[4] X. Shi, L, Zuo, S. B. Jo, K. Gao, F. Lin, F. Liu, A. K.-Y. Jen, *Chem. Mater.* **2017**, *29*, 8369.

[5] H. Yao, Y. Chen , Y. Qin, R. Yu, Y. Cui, B. Yang, S. Li, K. Zhang, J. Hou, *Adv. Mater.* **2016**, *28*, 8283.

[6] F. Liu, Z. Zhou, C. Zhang, J. Zhang, Q. Hu, T. Vergote, F. Liu, T. P. Russell, X. Zhu, *Adv. Mater.* **2017**, *29*, 1606574.

[7] Y. Li, J.-D. Lin, X. Che, Y. Qu, F. Liu, L.-S. Liao, S. R. Forrest, *J. Am. Chem. Soc.* **2017**, *139*, 17114.

[8] Z. Yao, X. Liao, K. Gao, F. Lin, X. Xu, X. Shi, L. Zuo, F. Liu, Y. Chen, A. K. Y. Jen, *J. Am. Chem. Soc.* **2018**, *140*, 2054.

[9] Z. Xiao, X. Jia, L. Ding, *Sci. Bull.* **2017**, *62*, 1562.

[10] J. Qu, Q. Zhao, J. Zhou, H. Lai, T. Liu, D. Li, W. Chen, Z. Xie, F. He, *Chem. Mater.* **2019**, *31*, 1664.

[11] J. Yuan, Y. Zhang, L. Zhou, G. Zhang, H.-L. Yip, T.-K. Lau, X. Lu, C. Zhu, H. Peng, P. A. Johnson, M. Leclerc, Y. Cao, J. Ulanski, Y. Li, Y. Zou, *Joule*, **2019**, *3*, 1.

[12] J. Yuan, T. Huang, P. Cheng, Y. Zou, H. Zhang, J. L. Yang, S.-Y. Chang, Z. Zhang, W. Huang, R. Wang, D. Meng, F. Gao, Y. Yang, *Nat. Commun*. **2019**, *10*, 570.

[13] J. Chen, G. Li, Q. Zhu, X. Guo, Q. Fan, W. Ma, M. Zhang, *J. Mater. Chem. A*, **2019**, *7*, 3745–3751.

[14]. F.-X. Chen, J.-Q. Xu, Z.-X. Liu, M. Chen, R. Xia, Y. Yang, T.-K. Lau, Y. Zhang, X. Lu, H.-L. Yip, A. K.-Y. Jen, H. Chen, C.-Z. Li, *Adv. Mater.* **2018**, *30*, 1803769.

[15]. H. Yao, Y. Cui, R.Yu, B. Gao, H. Zhang, J. Hou, *Angew. Chem. Int. Ed.* **2017**, *56*, 3045–3049.

[16]. R. Geng, X. Song, H. Feng, J. Yu, M. Zhang, N. Gasparini , Z. Zhang, F. Liu, D. Baran, W. Tang, *ACS Energy Lett.* **2019**, *4*, 763–770.

[17]. K. Wang, J. Lv, T. Duan, Z. Li, Q. Yang, J. Fu, W. Meng, T. Xu, Z. Xiao, Z. Kan, K.

Sun, S. Lu, *ACS Appl. Mater. Interfaces* **2019**, *11*, 6717−6723.
[18]. J. Lee, S.-J. Ko, M. Seifrid, H. Lee, B. R. Luginbuhl, A. Karki, M. Ford, K. Rosenthal, K. Cho, T.-Q. Nguyen, G. C. Bazan, *Adv. Energy Mater.* **2018**, *8*, 1801212.
[19] L. Zhang, K. Jin, Z. Xiao, X. Wang, T. Wang, C. Yi, L. Ding, *Mater. Chem. Front.* **2019**, *3*, 492-495.
[20]. T. Li, H. Zhang, Z. Xiao, J. J. Rech, H. Niu, W. You, L. Ding, *Mater. Chem. Front.* **2018**, *2*, 700-703.
[21] R. Ming, J. Wang, W. Gao, M. Zhang, J. Gao, W. Ning, Z. Luo, X. Liu, C. Zhong, F. Zhang, C. Yang, *Small Methods* **2019**, 1900280.
[22] Q. An, W. Gao, F. Zhang, J. Wang, M. Zhang, K. Wu, X. Ma, Z. Hu, C. Jiao and C. Yang, *J. Mater. Chem. A* **2018**, 6, 2468.
[23] Z. Zhou, W. Liu, Z. Zhang, F. Liu, H. Yan and X. Zhu, *Adv. Mater.* **2017**, 29, 1704510.
[24] S. Li, L. Ye, W. Zhao, S. Zhang, S. Mukherjee, H. Ade and J. Hou, *Adv. Mater.* **2016**, 28, 9423.
[25] S. Li, L. Ye, W. Zhao, S. Zhang, H. Ade and J. Hou, *Adv. Energy Mater.* **2017**, 7, 1700183.
[27] W. Zhao, D. Qian, S. Zhang, S. Li, O. Inganäs, F. Gao and J. Hou, *Adv. Mater.* **2016**, 28, 4734.
[28] Z. Zhang, M. Li, Y. Liu, J. Zhang, S. Feng, X. Xu, J. Song and Z. Bo, *J. Mater. Chem. A* **2017**, 5, 7776.
[29] B. Kan, H. Feng, X. Wan, F. Liu, X. Ke, Y. Wang, Y. Wang, H. Zhang, C. Li, J. Hou and Y. Shen, *J. Am. Chem. Soc.* **2017**, 139, 4929.
[30] N. Qiu, H. Zhang, X. Wan, C. Li, X. Ke, H. Feng, B. Kan, H. Zhang, Q. Zhang, Y. Lu and Y. Chen, *Adv. Mater.* **2017**, 29, 1604964.
[31] Y. Liu, Z. Zhang, S. Feng, M. Li, L. Wu, R. Hou, X. Xu, X. Chen and Z. Bo, *J. Am. Chem. Soc.* **2017**, 139, 3356.
[32] W. Liu, J. Zhang, Z. Zhou, D. Zhang, Y. Zhang, S. Xu and X. Zhu, *Adv. Mater.* **2018**, 30, 1800403.
[33] Y. Chen, T. Liu, H. Hu, T. Ma, J. Y. L. Lai, J. Zhang, H. Ade and H. Yan, *Adv. Energy Mater*. **2018**, 8, 1801203.
[34] W. Zhao, S. Li, H. Yao, S. Zhang, Y. Zhang, B. Yang, J. Hou, *J. Am. Chem. Soc.* **2017**, 139, 7148.
[35] F. Zhao, S. Dai, Y. Wu, Q. Zhang, J. Wang, L. Jiang, Q. Ling, Z. Wei, W. Ma, W. You, C. Wang, X. Zhan, *Adv. Mater.* **2017**, 29, 1700144.

[36] D. Liu, B. Yang, B. Jang, B. Xu, S. Zhang, C. He, H.Y. Woo, J. Hou, *Energy Environ. Sci.* **2017**, 10, 546.

[37] D. Xie, T. Liu, W. Gao, C. Zhong, L. Huo, Z. Luo, K. Wu, W. Xiong, F. Liu, Y. Sun, C. Yang, *Sol. RRL* **2017**, 1, 1700044.

[38] Y. Yang, Z.-G. Zhang, H. Bin, S. Chen, L. Gao, L. Xue, C. Yang, Y.F. Li, *J. Am. Chem. Soc.* **2016**, 138, 15011.

[39] L. Xue, Y. Yang, J. Xu, C. Zhang, H. Bin, Z. Zhang, B. Qiu, X. Li, C, Sun, L. Gao, J. Yao, X. Chen, Y. Yang, M. Xiao, Y, Li, *Adv. Mater.* **2017**, 1703344.

[40] S. Dai, F. Zhao, Q. Zhang, T.-K. Lau, T. Li, K. Liu, Q. Ling, C. Wang, X. Lu, W. You, X. Zhan, *J. Am. Chem. Soc.* **2017**, 139, 1336

[41] Z. Zheng, O.M. Awartani, B. Gautam, D. Liu, Y. Qin, W. Li, A. Bataller, K. Gundogdu, H. Ade, J. Hou, *Adv. Mater.* **2017**, 29, 1604241.

[42] Y. Li, L. Zhong, B. Gautam, H.-J. Bin, J.-D. Lin, F.-P. Wu, Z. Zhang, Z.-Q. Jiang, Z.-G. Zhang, K. Gundogdu, Y. Li, L.-S. Liao, *Energy Environ. Sci.* **2017**, 10, 1610.

[43] H. Yao, Y. Cui, R. Yu, B. Gao, H. Zhang, J. Hou, *Angew. Chem. Int. Ed.* **2017**, 56, 1.
